# tsRNA-GlyGCC promotes colorectal cancer progression and 5-FU resistance by regulating SPIB

**DOI:** 10.1186/s13046-024-03132-6

**Published:** 2024-08-17

**Authors:** Rong Xu, Ashuai Du, Xinpei Deng, Wei Du, Kaiying Zhang, Jianbo Li, Yingxue Lu, Xiaoli Wei, Qinglong Yang, Hailin Tang

**Affiliations:** 1 Department of Pathology, Changde Hospital, Xiangya School of Medicine, Central South University (The first people’s hospital of Changde city), Changde, Hunan 415000 China; 2 Department of Infectious Diseases, Guizhou Provincial people’s Hospital, Guiyang, Guizhou 550002 China; 3 State Key Laboratory of Oncology in South China, Guangdong Provincial Clinical Research Center for Cancer, Sun Yat-sen University Cancer Center, 651 Dongfeng East Road, Guangzhou, Guangdong 510060 China; 4 Department of Pathology, Xiangya Changde Hospital, Changde, Hunan 415000 China; 5 Department of General Surgery, Guizhou Provincial people’s Hospital, Guiyang, Guizhou 550002 China

**Keywords:** tsRNA, 5-FU resistance, CRC, SPIB, m^7^G, METTL1, JAK1/STAT6

## Abstract

**Background:**

tRNA-derived small RNAs (tsRNAs) are newly discovered non-coding RNA, which are generated from tRNAs and are reported to participate in several biological processes in diseases, especially cancer; however, the mechanism of tsRNA involvement in colorectal cancer (CRC) and 5-fluorouracil (5-FU) is still unclear.

**Methods:**

RNA sequencing was performed to identify differential expression of tsRNAs in CRC tissues. CCK8, colony formation, transwell assays, and tumor sphere assays were used to investigate the role of tsRNA-GlyGCC in 5-FU resistance in CRC. TargetScan and miRanda were used to identify the target genes of tsRNA-GlyGCC. Biotin pull-down, RNA pull-down, luciferase assay, ChIP, and western blotting were used to explore the underlying molecular mechanisms of action of tsRNA-GlyGCC. The MeRIP assay was used to investigate the N(7)-methylguanosine RNA modification of tsRNA-GlyGCC.

**Results:**

In this study, we uncovered the feature of tsRNAs in human CRC tissues and confirmed a specific 5’ half tRNA, 5’tiRNA-Gly-GCC (tsRNA-GlyGCC), which is upregulated in CRC tissues and modulated by METTL1-mediated N(7)-methylguanosine tRNA modification. In vitro and in vivo experiments revealed the oncogenic role of tsRNA-GlyGCC in 5-FU drug resistance in CRC. Remarkably, our results showed that tsRNA-GlyGCC modulated the JAK1/STAT6 signaling pathway by targeting SPIB. Poly (β-amino esters) were synthesized to assist the delivery of 5-FU and tsRNA-GlyGCC inhibitor, which effectively inhibited tumor growth and enhanced CRC sensitive to 5-FU without obvious adverse effects in subcutaneous tumor.

**Conclusions:**

Our study revealed a specific tsRNA-GlyGCC-engaged pathway in CRC progression. Targeting tsRNA-GlyGCC in combination with 5-FU may provide a promising nanotherapeutic strategy for the treatment of 5-FU-resistance CRC.

**Graphical Abstract:**

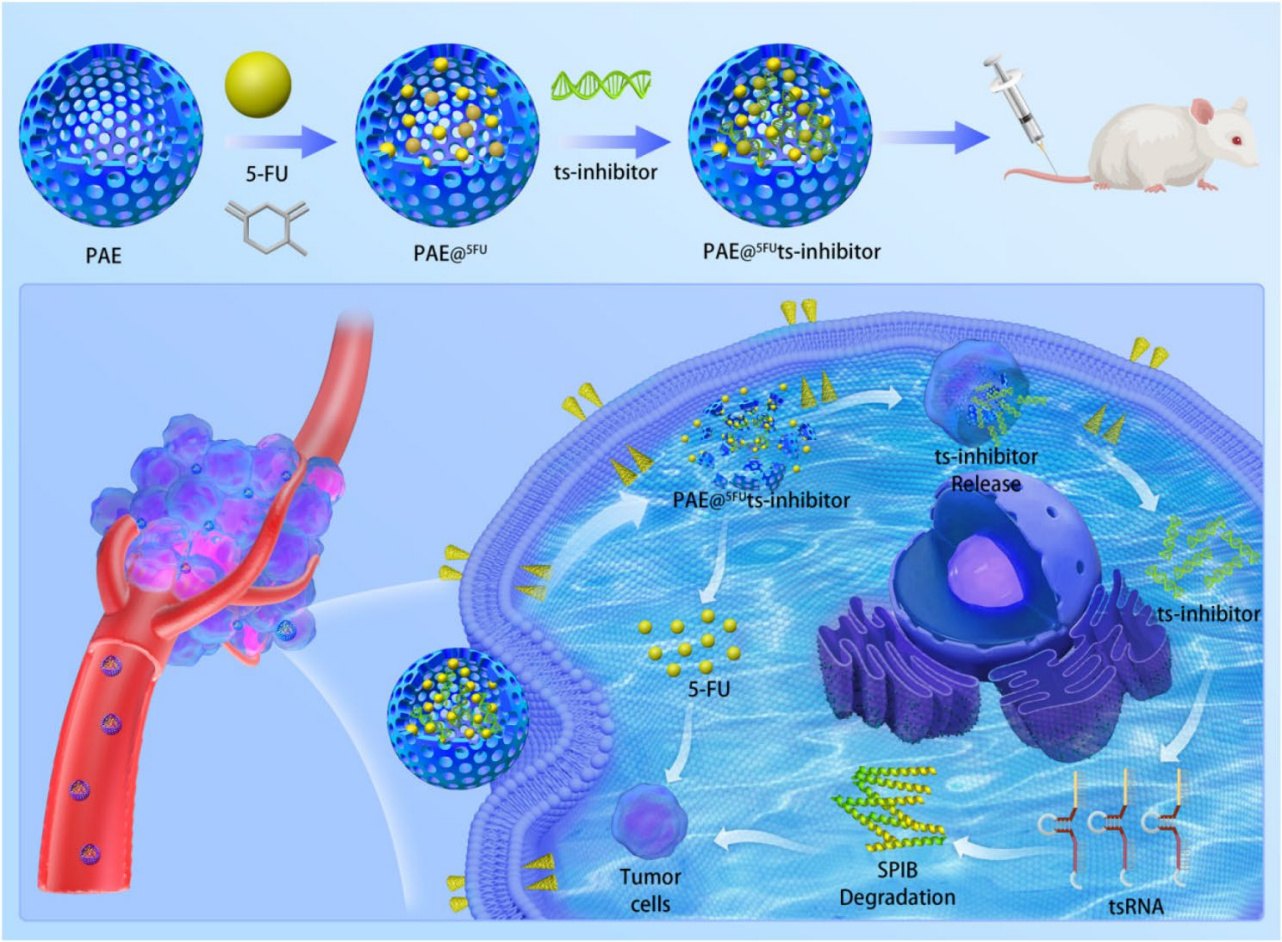

**Supplementary Information:**

The online version contains supplementary material available at 10.1186/s13046-024-03132-6.

## Introduction

Colorectal cancer (CRC) is the most common malignancy worldwide and ranks second in cancer-related mortality [1, 2].Surgery and chemotherapy are the primary treatments for CRC [3]. However, the prognosis of patients with CRC remains poor owing to high rates of metastasis and recurrence after therapy [4, 5]. Currently, chemotherapeutic drugs are the main treatment option for patients with progressive and metastatic CRC [6, 7]. 5-Fluorouracil (5-FU) is a widely used single agent or key component of systemic chemotherapy for CRC treatment [8, 9]. Although 5-FU or 5-FU-based combination chemotherapy regimens have beneficial effects, patients with advanced CRC still exhibit poor prognoses owing to acquired drug resistance [10, 11].The pathological mechanisms underlying CRC and 5-FU resistance should be thoroughly investigated to identify novel biomarkers or therapeutic targets [12].

To date, many studies have focused on non-coding RNAs, such as microRNAs (miRNAs), long non-coding RNAs, piwi-interacting RNA, and circular RNAs [13, 14]. However, a novel non-coding RNA, tRNA, which is a well-known carrier for amino acids, has gradually gained interest from researchers. tRNA can be cleaved into several fragments that are regarded as junk in cells. Recent studies revealed that these fragments play important roles in various cellular processes, including epigenetic regulation, mRNA stability repression, and translational inhibition [15–17]. Thus, these fragments have been identified as a new class of small non-coding RNAs called tRNA-derived small RNAs (tsRNAs). tsRNAs can be grouped into two types, tRNA halves and smaller tRNA fragments (tRFs), according to the enzyme recognition site and length [17]. A recent study demonstrated that tsRNAs are closely associated with tumorigenesis [18]. For example, 5’tiRNA-His-GTG promotes CRC progression by targeting LARS2 and modulating hippo signaling pathway [19]. tsRNA derived from tRNA-Glu, Asp, Gly, and Tyr can bind to Y-box binding protein 1, resulting in the stability of oncogenic transcripts [20]. Despite increasing interest in tsRNAs, their biogenesis and mechanisms remain largely unknown. Further studies are required to elucidate the functions and mechanisms of tsRNAs in physiological and pathological processes.

7-Methylguanosine (m^7^G) modification is a common modification, which is widely found in various molecules, such as mRNA 5’ cap structure, mRNA internal, pri-miRNA, transport RNA (tRNA), and ribosomal RNA (rRNA) [21, 22], and is involved in biological and pathological process [23]. Increasing evidence suggests that m^7^G plays a pivotal role in cancer development, drug resistance, and tumor therapy [23–25]. In general, the m⁷G modification occurs at position 46 of the tRNA variable region and then forms a tertiary base pair with C13-G22, resulting in the stabilization of the tRNA structure or regulation of mRNA translation [21]. However, researchers have found that m^7^G is present at positions other than 46. For example, chloroplast tRNA^Leu^(UAG) is modified with m^7^G at position 36 of the tRNA codon [26]. In addition, one study reported that m^7^G occurs at position 34 of the anticodon tRNA^Ser^(GCU) in starfish mitochondria [27]. In our study, we found that tRNA-GlyGCC has m^7^G at positions 29 and 45, mediated by methyltransferase-like protein-1 (METTL1), which mediates m^7^G tRNA modification for the stabilization of tRNA-GlyGCC and to promote the splicing and synthesis of tsRNA-GlyGCC.

Drug resistance is regarded as a key factor in the antitumor therapies of CRC [28–30]. Emerging evidence suggests that tsRNAs regulate drug resistance in multiple tumors [29, 31]. For example, tDR-0009 and tDR-7336, which are upregulated in triple-negative breast cancer cells, sustain the reactivity of interleukin-6, ultimately resulting in multidrug resistance through the activation of downstream pathways [32]. Another study indicated that tRF-30-JZOYJE22RR33 and tRF-27-ZDXPHO53KSN induce trastuzumab resistance in breast cancer cells with positive expression of HER2 [33]. Based on the multifunctional features of tsRNAs, it is worth investigating the underlying mechanisms of tsRNAs in 5-FU resistance in CRC.

## Materials and methods

### CRC samples

Matched adjacent normal and CRC tissues were collected from Guizhou Provincial People’s Hospital (Guizhou, China). Samples were diagnosed based on the clinical information of patients with CRC. All sample collection procedures were approved by the Ethics Committees of Guizhou Provincial People’s Hospital (approval number: 2023 − 285). The 10 samples (5 matched adjacent normal tissues and 5 CRC tissues) were used for RNA sequencing (RNA-seq) and 60 paired samples were used for further verification (qPCR and fluorescence in situ hybridization (FISH)). The evaluation criteria for 5-FU resistance in CRC based on Response Evaluation Criteria in Solid Tumors (recist) criteria [34]. The patients with disease progression (PD) are regarded as non-response CRC groups, complete response (CR) and partial response (PR) after chemotherapy treatment were regarded as response CRC groups.

### Preparation of PAE

Considering the critical role of tsRNAs in the tumorigenesis and drug resistance of CRC, PAE materials have been used as delivery vehicles for tsRNA-targeting inhibitors to provide potential therapies for CRC. PAE was synthesized as described previously [35].

### Drug encapsulation and in vitro release

The encapsulation efficiency of 5-FU in PAE was determined using a UV-Vis spectrophotometer. Briefly, 1 mg PAE was dispersed in 1 mL of distilled water to extract 2 mg 5-FU, and then gently shaken at 37 °C for 12 h. The filtrates were diluted with methanol at a ratio of 1:10 and the solution was analyzed at 266 nm. The encapsulation and loading efficiencies were calculated as previously described [36].

### Preparation of PAE-inhibitor complex

To prepare the PAE**@**^**5−FU**^ ts-inhibitor complex, 1 mg/mL PAE**@**^**5−FU**^ was mixed with the inhibitor solution in PBS (pH = 6.0) and shaken for 1 h to complete the binding of the tsRNA inhibitor to the PAEs via electrostatic interactions. The weight ratio of the tsRNA inhibitor to PAE of 50:1 was used as the saturated concentration of the inhibitor solution. PAE binding to the NC inhibitor was abbreviated as PAE-NC inhibitor and it acted as a control tsRNA.

### Characterization

The particle morphology was examined using transmission electron microscopy (Tecnai G2 F20, USA). A Malvern Zetasizer Nano ZS 90 zeta potential analyzer (UK) was used to analyze the size distribution and surface charge.

### Subcellular location of nanoparticle

For the investigation of subcellular location, HCT116/R cells were seeded in 6-well plates and cultured in a 5% CO_2_ incubator at 37^o^C. Cells were treated with PBS and fixed with 4% paraformaldehyde for 10 min. The cells were then treated with the free tsRNA inhibitor-cy5 or PAE@^5−FU^ts-inhibitors-cy5, and washed with PBS three times. DAPI was used to display nuclear morphology, and fluorescent images were recorded using confocal laser scanning microscopy.

### Cell culture

Human CRC cell lines (HCT116, HT29, SW480, and SW620), HCT116 5-FU resistance cell, and mouse colorectal cancer cell lines MC38 and MC38-LUC were purchased from Shanghai Model Organisms Company. All cell lines were cultured in RPMI1640 with 10% FBS (Inner Mongolia Opcel Biotechnology Co., Ltd.). The siRNA for specific inhibition of JAK1 was purchased from RiboBio Co., Ltd.

### Construction of 5-FU resistance cell model

HCT116 and SW480 cell lines were continuously treated with a gradually increasing concentration of 5-FU rising from 10^− 8^M to 10^− 4^M initially. The resistance index (RI, IC50 of WT cells /IC50 of the resistance cells) > 10 was defined as 5-FU resistance cell lines. The details of building resistance cell model as previously described [37].

### tsRNA sequencing

Five matched adjacent normal tissues and five CRC tissues were used for tsRNA sequencing, which was performed by RiboBio Co., Ltd.

### RNA and qPCR

Total RNA was extracted using the TRIzol reagent. A total of 2 µg RNA was used to synthesize cDNA using the reverse transcription kit (Thermo Fisher, USA). RNA levels were detected using qPCR. RNA expression was normalized to those of GAPDH and tsRNA expression levels were normalized to those of U6. The 2^−ΔΔCt^ method was used to analyze the relative RNA expression. All primer sequences are showed in Table 1.

**Table 1:** Primer sequence

Gene name	Primer sequence
tRF-31-P4R8YP9LON4VD	F: CATGGGTGGTTCAGTGGTAGAATT R: GTCGTATCCAGTGCAGGGTCCGAGGT
	RT: GTCGTATCCAGTGCAGGGTCCGAGGTATTCGCACTGGATACGACGGCGAG
tRF-28-Q99P9P9NH50E	F: GCTTCTGTAGTGTAGTGGTTAT R: GTCGTATCCAGTGCAGGGTCCGAGGT
	RT: GTCGTATCCAGTGCAGGGTCCGAGGTATTCGCACTGGATACGACAACGTG
tRF-32-J87383RPD9W1P	F: ATGCATGGGTGGTTCAGTGGT R: GTCGTATCCAGTGCAGGGTCCGAGGT
	RT: GTCGTATCCAGTGCAGGGTCCGAGGTATTCGCACTGGATACGACCACCGC
tRF-27-OIQO4QPRJW3	F: TCGAGAACTGCTAACTCATGCC R: GTCGTATCCAGTGCAGGGTCCGAGGT
	RT: GTCGTATCCAGTGCAGGGTCCGAGGTATTCGCACTGGATACGACAGACAT
U6	F: CTCGCTTCGGCAGCACATATACT R: GTCGTATCCAGTGCAGGGTCCGAGGT RT: AAAATATGGAACGCTTCACGAATTTG
SPIB	F: CCCTATGAAGCCTTCGACCC R: CCAGCAGGAACTGGTACAGG
JAK1	F: GGTAGATGGCTACTTCCGGC R: TGCACCTGCTCAGACTTCTC
JAK2	F: CCGATCTGTGTAGCCGGTTT R: GTAAGGCAGGCCATTCCCAT
JAK3	F: CCTTCGAAAGTCCAGGGTCC R: CCAGAGCAAAGAGGGAGTGG
STAT1	F: TGTGAAGTTGAGAGATGTGAATGA R: TTGGAGATCACCACAACGGG
STAT3	F: ACCCACTCCTTGCCAGTTGT R: GGCCACTTGATCCCAGGTT
STAT4	F: GAGACCAGCTCATTGCCTGT R: CAATGTGGCAGGTGGAGGAT
STAT6	F: CATTTGGTACAACGTGTCAACCA R: TGTGGCAGGTG GAGGATTATTA
GAPDH	F: GAGATCCCTCCAAAATCAAGTG R: GAGTCCTTCCACGATACCAAAG

### Western blotting

The total protein was extracted using RIPA with a 1% protease inhibitor cocktail for 15 min. Subsequently, the cell lysate was centrifuged for 30 min at 4^o^C and 13,000 rpm. The protein concentration was determined using the BCA test kit. Western blotting was performed as previously described [38]. Relevant primary antibodies: GAPDH (CST #92,310), SPIB (15768-1-AP, Proteintech), BAX (380,709, zenbio), BCL2 (R23309, zenbio), EphB2 (83277-1-RR, Proteintech), L1CAM (R381761, zenbio), LGR5 (R380973, zenbio). STAT6 (ab32520, abcam), JAK1 (ab125051, abcam), METTL1 (ab271063, abcam), CD44 (15675-1-AP, Proteintech), and CD133 (18470-1-AP, Proteintech); secondary antibodies: anti-rabbit IgG antibody (ab288151) and anti-mouse IgG antibody (ab205719).

### Colony formation assay

CRC cells were added to 6-well plates at a density of 1 × 10^3^ cells/well, and 2 weeks later, colonies were stained with crystal violet for 30 min. The samples were washed with water and dried at 30^o^C.

### Transwell assay

CRC cells were added to the upper chamber with RPMI 1640 (without FBS), and the lower chamber was filled with the RPMI 1640 supplemented with 10% FBS. After 48 h, cells were stained with 1% crystal violet dissolved in methanol for 15 min. The cells were observed under a light microscope (Motic, China).

### CHIP assay

ChIP assays were performed using the ChIP kit (Sigma-Aldrich), according to the manufacturer’s protocol. The CHIP assay was performed after flag-tagged SPIB was transfected into HCT116 cells. The primers used are listed in Table 1.

### Luciferase reporter assay

STAT6 promoter regions, including − 2000 bp upstream of the transcription start site or mutant sequences, were inserted into the pGL3-enhancer vector. Firefly and Renilla luciferase activity was measured using a dual-luciferase system.

### Bioinformatics analysis

The expression array data GSE126092 and GSE115513 were downloaded from the Gene Expression Omnibus Database for SPIB expression and GSEA analyses. Sanger box (http://sangerbox.com/) was used to analyze the expression of METTL1 and SPIB. The JASPER and ALGGEN PROMO online tools were used to identify a consensus SPIB binding site in the promoter region of STAT6. Another online tool (http://lin-group.cn/server/iRNA-m7G/) was used for m^7^G site analysis of tRNA and tsRNA.

### Immunofluorescence staining

Immunofluorescence staining was conducted as previously described [39]. Briefly, the cells were fixed with methanol for 5 min. A total of 5% BSA was used to block non-specific proteins and was subsequently incubated with relevant primary antibodies overnight at 4^o^C, and incubated with secondary antibodies labelled with Cy5 or FITC for 2 h at room temperature. The cell’s nucleus was stained with DAPI solution. Laser scanning confocal microscopy was used for image acquisition.

FISH assay was used to detect tsRNA expression and localization in tissue specimens. Briefly, the slides were treated with 100 µL pre-hybridization buffer at 37^o^C for 30 min and incubated with FISH probes in dark at 37^o^C overnight. Subsequently, they were washed with wash buffers I, II, and III at 42^o^C and once with PBS at 25^o^C. Lastly, they were counter-stained with DAPI. The tsRNA FISH probes were designed and synthesized by RiboBio Co., Ltd. Images were captured using a fluorescence microscope (Leica).

### Flow cytometry assay

An Annexin V-FITC/propidium iodide double staining kit (Vazyme, Nanjing, China) was used to test cell apoptosis, the cells were stained with Annexin V-FITC, and propidium iodide flow cytometer (Cytek Biosciences, USA) was used to measure the cell apoptosis ratio.

### MeRIP assay

Verification of the modification site of m^7^G on tRNA was conducted by Aksomics Co., Ltd. Briefly, RNA was heated to 65^o^C for 5 min. The IP reaction system contained 27 µL of the sample, 60 µL IP buffer (50 mM tris pH 7.4, 750 mM NaCL 0.5% NP-40), 3 µL RNase inhibitor, 2 µL m^7^G anti-7-methylguanosine antibody, and 210 µL RNase free water at 4^o^C for 2 h. Subsequently, IP reaction system was incubated with 20 µL rabbit IgG magnetic beads overnight at 4^o^C, 200 µL elution buffer was readded, along with 4 uL proteinase and 2 uL RNAase inhibitor and was incubated for 1 h at 50^o^C, after being washed with IP buffer three times. The supernatant was used for RNA extraction and binding-fragment enrichment analyses.

### Murine models of colon cancer

All animal experiments were approved by Guizhou Provincial People’s Hospital (approval number: 2023 − 122). This was the first animal study to evaluate the antitumor effects of tsRNA inhibitors. Four-week-old BALB/c nude mice were randomly assigned to one of the four groups: NC agomir, tsRNA agomir, NC antagomir, and tsRNA antagomir. Mice were injected subcutaneously with HCT116/R CRC cells (5 × 10^6^). When the tumor reached 50 mm^3^, they were treated with 40 µg tsRNA agomir or NC antagomir/tsRNA antagomir every 3 days using intertumoral injection.

To evaluate the tumor targeting ability of PAE@^5−FU^ts-inhibitors, mice with subcutaneous tumors were injected with tsRNA inhibitor-cy5 and PAE@^5−FU^ts-inhibitors-cy5 (40 µg per mouse equivalent) through the tail vein. At 6, 24, and 48 h post-injection, the IVIS Spectrum in vivo imaging system (PerkinElmer) was used to measure the fluorescence intensity of the mice, and ex vivo fluorescent images were obtained using the same measurement system.

The antitumor effect of PAE@^5−FU^ts-inhibitor. HCT116/R cells (5 × 10^6^) were subcutaneously injected into 4-week-old BALB/c nude mice. Since tumor volume had reached to approximately 200 mm^3^, mice were divided into different groups (*n* = 4 in each group), and intravenously treated with PBS (50 µL, PBS), 5-FU (50 µL, 5-FU:25 mg/kg per mouse), PAE@^5−FU^NC-inhibitor (50 µL, 5-FU:25 mg/kg per mouse), NC inhibitor (40 µg per mouse), PAE@^5−FU^ts-inhibitor (50 µL, 5-FU:25 mg/kg per mouse), and tsRNA-inhibitor (40 µg per mouse). The tumor volume and weight were measured at different time points. The tumor, heart, liver, spleen, lungs, and kidneys of the mice were treated with a 4% paraformaldehyde solution. The tissue slices were stained with hematoxylin and eosin and was observed using an optical microscope.

### Mouse lung metastasis model

MC38-LUC cells were used as a pulmonary metastatic tumor model. 200 µL of PBS containing 2 × 10^6^ MC38-LUC cells were injected into BALB/c nude mice via the tail vein. Two weeks after tumor cell injection, the mice were randomly assigned to different groups: PBS, 5-FU, PAE@^5−FU^NC-inhibitor, PAE@^5−FU^ inhibitors (*n* = 6 mice per group), and the weights of the mice were monitored during the experimental period. PET/CT was performed to evaluate tumor growth. After the intravenous injection of ^18^F-FDG, the mice were anesthetized and placed in a chamber. SUVmax of the tracer was determined according to a previously described method [35].

### Statistical analysis

All results were assessed by SPSS 20.0 and were expressed as means ± SD. Variance analysis was used to evaluate whether the data conforms to normal distribution and displayed by a QQ-plot. Student’s t-test was used to determines whether there is a statistically significance between two groups, which the data in two groups is continuous normally distributed variable, and satisfy normally distribution [40]. Unequal variances are assumed between two groups, then Wilcoxon-Mann-Whitney test was used for statistically analysis. The Chi-square test is a non-parametric statistic, which was used to analyze the correlation between the expression of tsRNA-GlyGCC and SPIB and clinical pathological information. Visualization of charts was done using the GraphPad Software. Correlations between tsRNAs and SPIB were analyzed using Pearson’s correlation analysis. Statistical significance was set at *P* < 0.05.

## Results

### tsRNA-GlyGCC is upregulated in CRC tissues

**Fig. 1:**
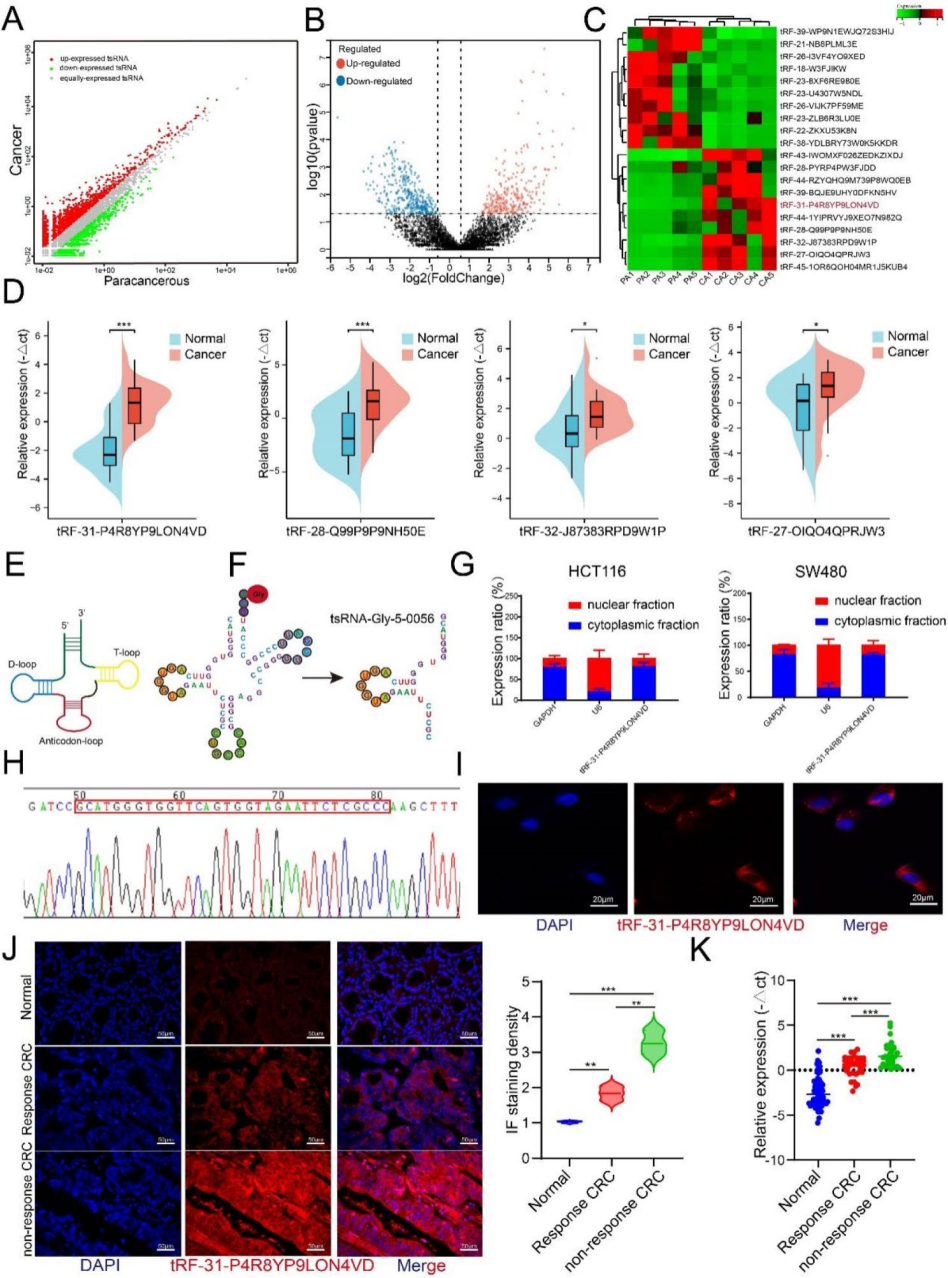
tsRNA-GlyGCC is overexpressed in CRC tissues. **A**-**C**. The difference of tsRNA profiles, volcano plot and heat map displaying differentially expressed tsRNA between five pairs of CRC and corresponding normal tissues; **D**. tsRNA-GlyGCC was significantly upregulation in CRC tissues; **E**. The structure of tRNA; **F**. tsRNA-GlyGCC was originated from tRNA-Gly-GCC; **G**. tsRNA-GlyGCC was mainly located in cell cytoplasm; **H**. The product of tsRNA-GlyGCC was confirmed by Sanger Sequencing; **I**. tsRNA-GlyGCC was located in cell cytoplasm by using IF assay; **J**. FISH assay was used for tsRNA-GlyGCC detection; **K**. qPCR was conducted to examine the expression of tsRNA-GlyGCC in normal, response CRC, and non-response CRC tissues, *p < 0.05, **p < 0.01, ***p < 0.01. All data are representative of at least three independent experiments and are presented as the means ± SD

To investigate the role of tsRNAs in CRC tumorigenesis, we performed RNA-seq of the total RNA obtained from the five paired CRC and paracancerous tissues. Differences in tsRNA profiles, volcano plots, and heat maps are shown in Fig. 1A-C. Based on differential expression levels of dysregulated tsRNAs (|fold change| ≥ 2 and *p*-value < 0.05), we selected four significantly up-regulated tsRNA for further validation by using qPCR in five paired CRC tissues and corresponding normal tissues. The results showed that tsRNA-GlyGCC, tRF-28-Q99P9P9NH50E, tRF-32-J87383RPD9W1P, and tRF-27-OIQO4QPRJW3 were significantly upregulated in CRC tissues (Fig. 1D).

tsRNAs originate from tRNA, which have a classical cloverleaf structure with three loops (Fig. 1E). tsRNA-GlyGCC originated from tRNA-Gly-GCC (Fig. 1F) and were mainly located in the cytoplasm of the CRC cells (Fig. 1G). We used stem-loop RT-PCR to detect tsRNA as previously described [19]. Sanger sequencing was used to detect the qPCR products, and the sequences matched perfectly (Fig. 1H). tsRNA-GlyGCC was significantly upregulated in the CRC tissues and was mainly located in the cytoplasm (Fig. 1I). qPCR was used to detect the expression of tsRNA-GlyGCC in CRC cells and normal colon epithelial cells, and the results showed that tsRNA-GlyGCC was significantly upregulated in CRC cell lines (figure S1A). Subsequently, FISH was used to detect the expression of tsRNA-GlyGCC in CRC and the corresponding normal tissues. The results showed that tsRNA-GlyGCC was upregulated in CRC 5-FU non-responsive tissues compared to that in CRC 5-FU responsive tissues (Fig. 1J). The level of tsRNA-GlyGCC was significantly upregulated in non-response CRC tissues compared with response CRC tissue by using qPCR methods (Fig. 1K). In addition, high tsRNA-GlyGCC expression was positively correlated to tumor metastases (Table 2).

**Table 2:** Associations between the expression levels of tsRNA and the clinicopathological characteristics of 60 CRC patients

Characteristics	tsRNA expression		χ^2^-value	*p*-value
	High (n = 30)	Low (n = 30)		
**Age, years**				
≥ 60 60 60	18	10	3.674	0.055
< 60	12	20		
**Gender**				
Male	22	24	0.461	0.497
Female	8	6		
**Tumor size(cm)** **Single** **Multiple**				
≤ 5	9	11	9.909	0.173
> 5	23	17		
**Tumor metastasis**				
Absent	13	25	6.461	0.011*
Present	15	7		
**TNM stage**				
I-II	16	21	2.018	0.155
III-IV	14	9		
**Tumor differentiation**				
I-II	20	24	1.540	0.215
III-IV	10	6		

**χ**^**2**^ test was used to test the association between two categorical variables

*Statistically significant

### tsRNA-GlyGCC plays an oncogenic role in vitro

**Fig. 2:**
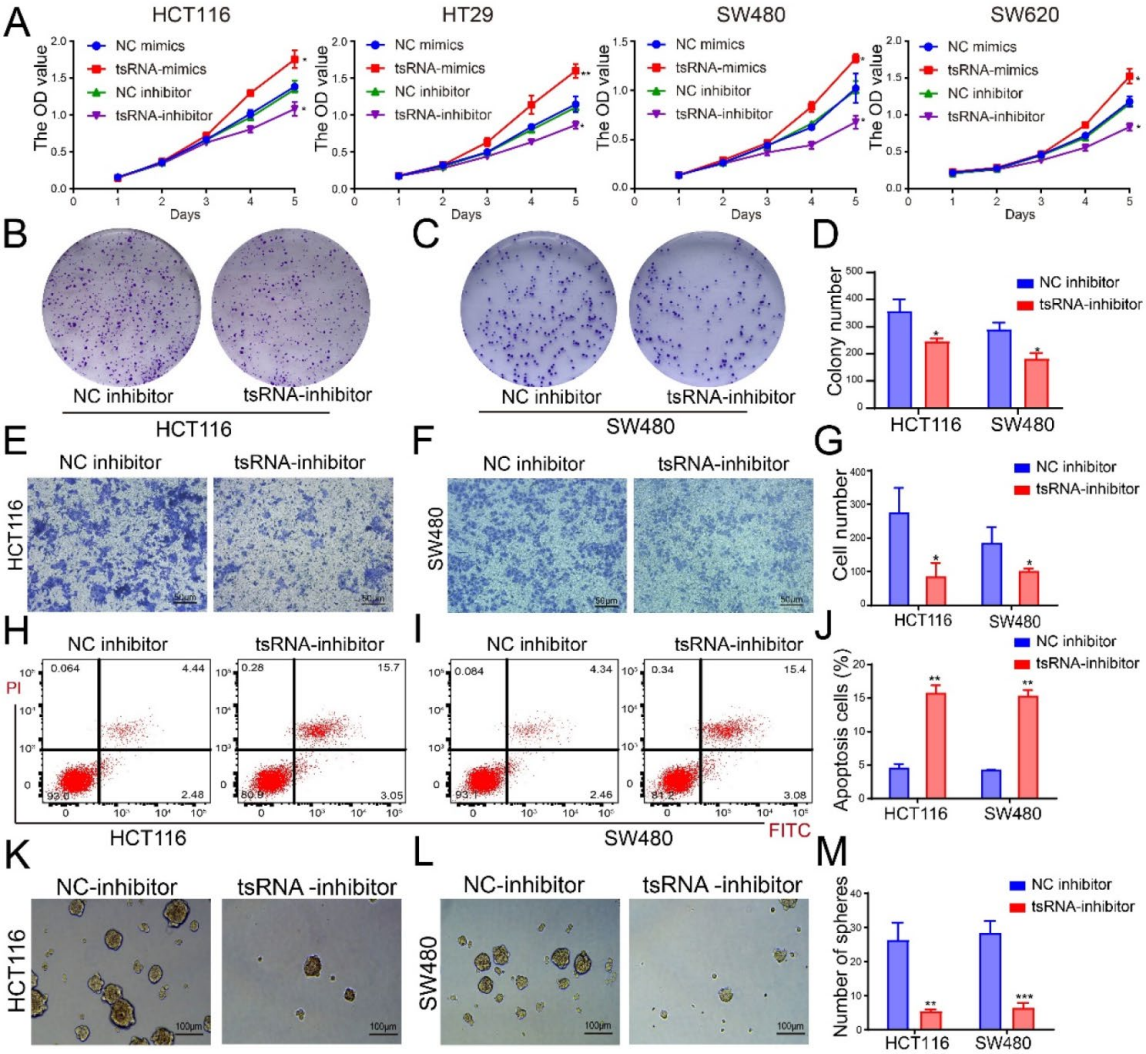
tsRNA-GlyGCC promote cell proliferation and reduce cell apoptosis in CRC. **A**.The tsRNA-GlyGCC inhibitor significantly decrease cell proliferation, while tsRNA-GlyGCC mimics promote cell reproductive capacity; **B**-**D**. tsRNA-GlyGCC inhibitor reduced anchorage-dependent growth in 5-FU resistance CRC cells; **E**-**G**. tsRNA-GlyGCC inhibitor reduced cell migration using transwell assays; **H**-**J**. tsRNA-GlyGCC inhibitor induced apoptosis using FACS analysis. **K**-**M**. tsRNA-GlyGCC inhibitor decreased the growth of cell spheroids. *p < 0.05, **p < 0.01, ***p < 0.01. All data are representative of at least three independent experiments and are presented as the means ± SD

tsRNA-GlyGCC is upregulated in CRC tissues and cell lines, and tsRNA-GlyGCC specific inhibitor could significantly reduce the expression of tsRNA-GlyGCC (Figure S1 B). The effects of tsRNA-GlyGCC inhibitors and mimics on cell proliferation were examined. As shown in Fig. 2A, the tsRNA-GlyGCC inhibitor significantly reduced cell proliferation, whereas the mimic promoted cell proliferation. Furthermore, the colonic formation assay indicated that the tsRNA-GlyGCC inhibitor markedly decreased the number of colonies (Fig. 2B-D) and migration (Fig. 2E-G). Flow cytometry analysis showed that the tsRNA-GlyGCC inhibitor increased apoptosis in CRC cells (Fig. 2H-J). Additionally, WB results indicated that the expression of BCL2 was reduced in tsRNA-GlyGCC inhibitor groups, while the expression of BAX was elevated in tsRNA-GlyGCC inhibitor groups (Figure S1C-E). tsRNA-GlyGCC is associated with tumor metastasis, therefore we assessed the antitumor efficacy of the tsRNA-GlyGCC inhibitor in CRC cell spheroids, the number and size of cell spheroids were remarkedly reduced by tsRNA-GlyGCC inhibitor (Fig. 2K-M). The qPCR results showed that tsRNA-GlyGCC was highly expressed in the spheroids (Figure S1 F). Moreover, western blot results indicated that the tsRNA-GlyGCC inhibitor reduced the protein levels of the CD44 and CD133 stem cell markers (Figure S1G-H), and more specific CSC-related markers L1CAM, EphB2 and LGR5 (Figure S1I-J).

### tsRNA-GlyGCC promote 5-FU resistance of CRC in vivo

**Fig. 3:**
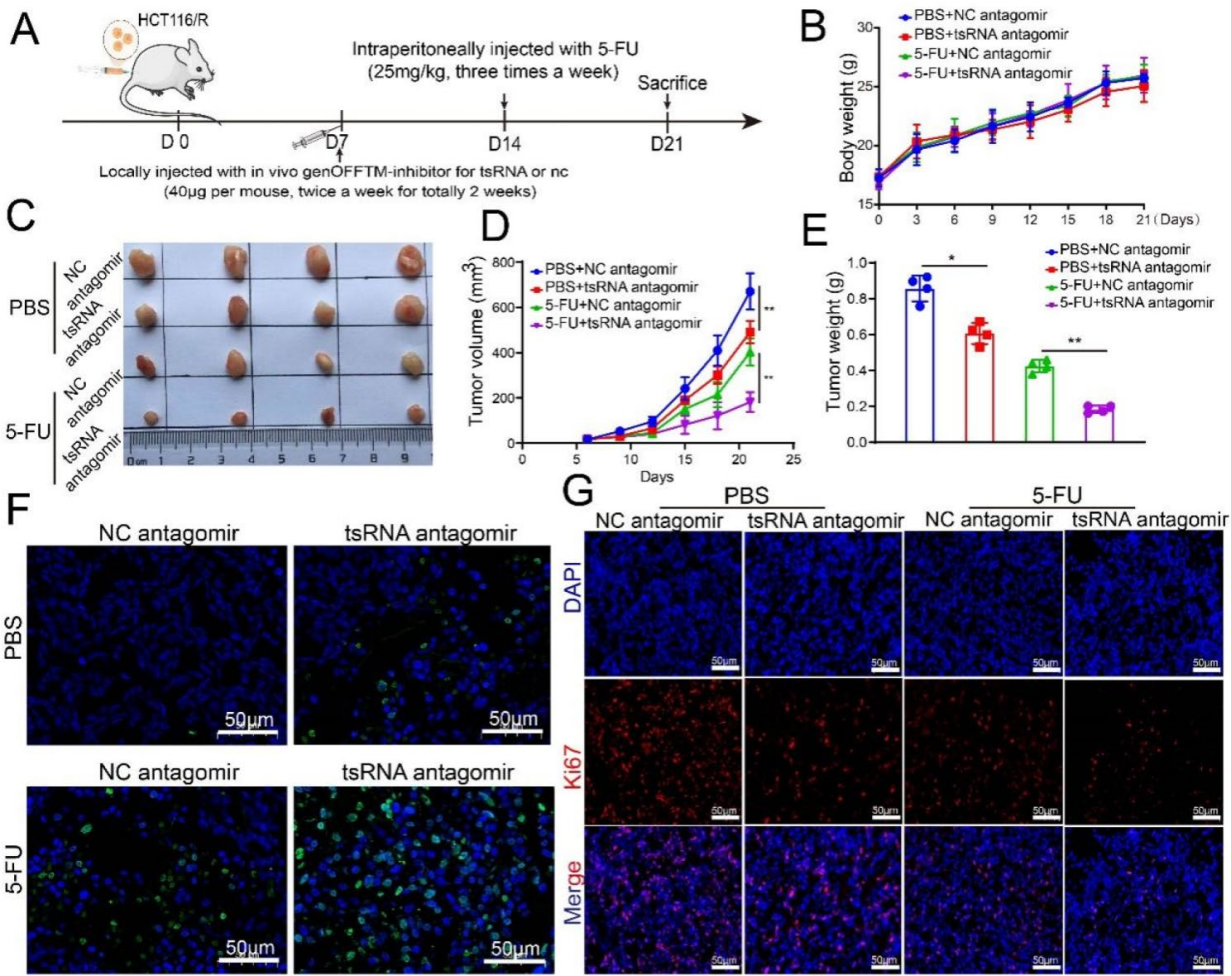
tsRNA-GlyGCC plays an oncogenic role in vivo. **A**. Flow chart of animal experiment; **B**. Body weight of mice in different groups; **C**. Representative photos of tumours; **D**: Tumor growth measured using a line chart; **E**. Tumor weights in different groups; **F**. Tunnel assay was used to detect cell apoptosis in different groups; **G**. Protein level of Ki67 in tumor tissues in different groups. *p < 0.05, **p < 0.01. All data are representative of at least three independent experiments and are presented as the means ± SD

The presence of cancer stem cells was closely associated with drug resistance. KEGG analysis revealed that the target genes of tsRNA-GlyGCC were enriched in cancer, specifically in JAK-STAT and PI3K-AKT signaling pathways (Figure S2A). We examined the role of tsRNA-GlyGCC in 5-FU resistance cells. Firstly, the CRC 5-FU resistance cell lines were established as previously described [37], and calculated the resistance indexes. The HCT116 and SW480 5-FU-R cells had resistance indexes of more than 100 at the end of 5-FU induction (Figure S2B-C). We found that tsRNA-GlyGCC was elevated in 5-FU resistance cell lines (Figure S3A-D) through qPCR and FISH assays, and the tsRNA-GlyGCC inhibitor significantly decreased inhibitory concentration (IC50) value in HCT116/R and SW480/R cells (Figure S3E-F), indicating the potential role of tsRNA-GlyGCC in CRC 5-FU resistance. To address the role of tsRNA-GlyGCC in vivo, HCT116/R cells were used to establish a xenograft tumor model in nude mice, which were then treated with NC agomir/antagomir or tsRNA-GlyGCC agomir/antagomir via local injections four times twice a week. Fourteen days after the subcutaneous injection, nude mice were intraperitoneally injected with 5-FU (Fig. 3A). Body weights of the four groups were not significantly different (Fig. 3B). However, the tsRNA-GlyGCC antagomir reduced tumor growth as observed by assessing tumor weight and volume, and the synergistic effects of the tsRNA-GlyGCC antagomir and 5-FU significantly reduced the tumor weight and volume (Fig. 3C-E). Moreover, the tsRNA-GlyGCC antagomir decreased the expression of Ki67 in tumor tissues and increased apoptosis (Fig. 3F-G, Figure S3G-H).

### tsRNA-GlyGCC directly targets SPIB

**Fig. 4:**
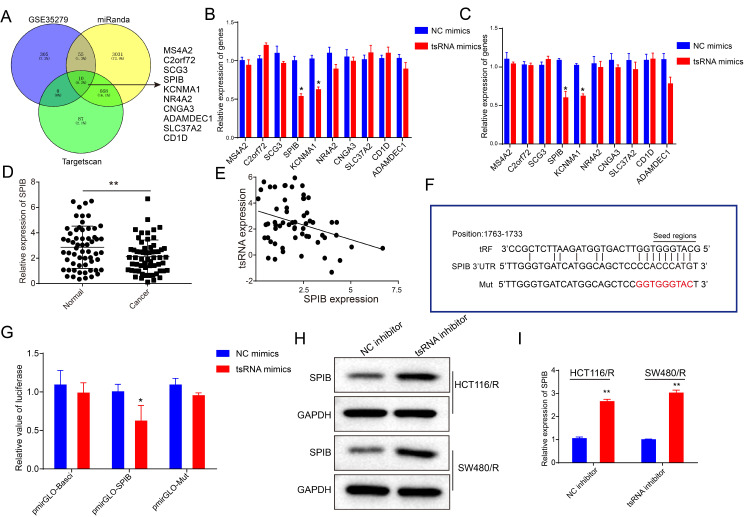
tsRNA-GlyGCC directly targets SPIB. **A**. Identification of potential target genes of tsRNA-GlyGCC; **B**-**C**. The mRNA expression in cells transfected with tsRNA-GlyGCC mimics; **D**. The mRNA level of SPIB in CRC and adjacent normal tissues; E. Correlation analysis between SPIB and tsRNA-GlyGCC; **F**. The binding site between SPIB and tsRNA-GlyGCC; **G**. Luciferase assay was used to check the luciferase activity of wide type and mut type groups; **H**-**I**. WB assay was used to detect the protein level of SPIB when transfected with tsRNA-GlyGCC inhibitor. *p < 0.05, **p < 0.01. All data are representative of at least three independent experiments and are presented as the means ± SD

We investigated the molecular mechanisms associated with tsRNA-GlyGCC in CRC progression. TargetScan and miRanda were used to predict potential target genes of tsRNA-GlyGCC. Ten potential target genes were identified in TargetScan and miRanda and were significantly downregulated in GSE35279 (Fig. 4A). The TCGA datasets indicated that SPIB was significantly downregulated in CRC compared to the corresponding normal tissues (Figure S4A). The Gene Expression Omnibus database showed that SPIB was downregulated in CRC tissues compared to normal tissues in GSE126092 (Figure S4B). The HPA database demonstrated that the protein level of SPIB was lower in colon cancer tissues than in normal colonic epithelial tissues (Figure S4C). Moreover, patients with low SPIB levels had shorter DFS than those with high SPIB (Figure S4D). These results demonstrate that SPIB may play an anticancer role in CRC.

To confirm the predicted target genes, tsRNA-GlyGCC mimics were overexpressed in CRC cells. The results showed that SPIB mRNA was downregulated in both HCT116 and SW480 cells (Fig. 4B-C). In addition, SPIB was significantly downregulated in CRC tissues compared to adjacent normal tissues (Fig. 4D) and negatively correlated with tsRNA-GlyGCC (*R*=-0.3292, *p* = 0.0102) (Fig. 4E). Clinicopathological analysis demonstrated that low SPIB expression was associated with a lower grade and differentiation of CRC (Table 3). The SPIB binding site in tsRNA-GlyGCC is shown in Fig. 4F. Luciferase assays were used to detect the luciferase value in different groups, tsRNA-GlyGCC mimics could bind to a wide range of SPIB 3’UTR and decrease the luciferase value. As shown in Fig. 4G, the SPIB protein levels increased in the indicated cells transfected with the tsRNA-GlyGCC inhibitor (Fig. 4H-I).

**Table 3:** Associations between the expression levels of SPIB and the clinicopathological characteristics of 60 CRC patients

Characteristics	SPIB expression		χ^2^-value	*p*-value
	High (n = 30)	Low (n = 30)		
**Age, years**				
≥ 60 60 60	17	11	2.411	0.121
< 60	13	19		
**Gender**				
Male	21	25	0.082	0.775
Female	7	7		
**Tumor size(cm)** **Single** **Multiple**				
≤ 5	10	12	0.287	0.592
> 5	20	18		
**Tumor metastasis**				
Absent	12	10	11.650	0.124
Present	13	25		
**TNM stage**				
I-II	17	10	5.511	0.022*
III-IV	11	22		
**Tumor differentiation**				
I-II	15	21	4.922	0.027*
III-IV	17	7		

**χ**^**2**^ test was used to test the association between two categorical variables

* Statistically significant

### SPIB regulates JAK1/STAT6 signaling pathway through inhibition of STAT6 transcription

**Fig. 5:**
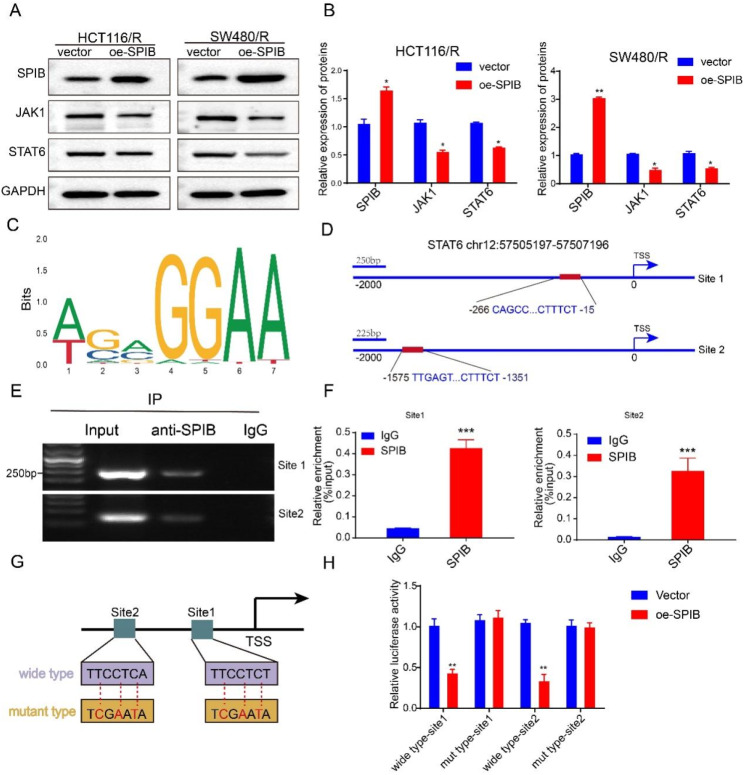
SPIB is a transcriptional repressor of STAT6. **A**-**B**. WB assay was used to detect the protein level of JAK1/STAT6; C. The binding sites of SPIB in STAT6 promoter region by using JASPAR database; **D**. Schematic diagrams of promoters of STAT6; **E**-**F**. ChIP assay was used to verify that SPIB could bind to STAT6 promoter region; **G**. Construction dual-luciferase reporter plasmids based on the binding sites of SPIB; **H**. Luciferase assay was used to detect luciferase activity in HCT116 cells overexpressing SPIB after transfection of pGL enhancer plasmids containing wide type or mut type STAT6 promoter. *p < 0.05, **p < 0.01, ***p < 0.001. All data are representative of at least three independent experiments and are presented as the means ± SD

SPIB is a member of the E-twenty-six transcription factor family, which can suppress gene activation and functions as a tumor suppressor gene in cancer [41]. In this study, we screened for possible genes that may be pre-transcriptionally regulated by SPIB, using JASPER, TRANSFAC, and MotifMap websites. We then detected JAKs and STATs genes in SPIB-overexpressing CRC cells and found that JAK1 and STAT6 were significantly downregulated (Figure S5A-B). Subsequently, we found that the protein levels of JAK1 and STAT6 were downregulated in SPIB-overexpressing cells (Fig. 5A-B), indicating a regulatory relation between SPIB and JAK1/STAT6 at the pre-transcriptional level. Interestingly, the JASPER website predicted binding sites for SPIB in the STAT6 promoter region (Fig. 5C). HCT116 cells were transfected with pCDNA/flag-SPIB, and anti-FLAG and IgG antibodies were used for ChIP assays. Furthermore, the STAT6 promoter region fragments, including the wild-type and mut-type sites 1 and 2 with the highest binding score, were amplified (Fig. 5D). As shown in Fig. 5E-F, SPIB was directly bound to the STAT6 promoter region. The promoter region of the binding sites was inserted into the pGL3-enhancer plasmids (Fig. 5G). SPIB overexpression suppressed luciferase activity in these regions of the STAT6 promoter (Fig. 5H). Next, JAK1 siRNA was used to conform this pathway involved in tsRNA-GlyGCC regulation of CRC carcinogenesis (figure S5C). As showed in figure S5D-E, tsRNA-GlyGCC mimics could increase expression of JAK1/STAT6, while JAK1 siRNA reverse the induction of JAK1/STAT6 by tsRNA-GlyGCC mimics. Additionally, IHC was used to detect the expression intensity of SPIB and STAT6 in xenograft tumors described in Fig. 3. The expression of SPIB was increased in tsRNA antagomir groups, and significantly increased in 5-FU + tsRNA antagomir groups compared with 5-FU + NC antagomir or tsRNA antagomir groups (Figure S5F). On the contrary, the expression of STAT6 was sharply decreased in 5-FU + tsRNA antagomir groups (Figure S5G).

### METTL1 regulates tsRNA-GlyGCC expression through m^7^G modification on 5’tRNA-Gly-GCC

**Fig. 6:**
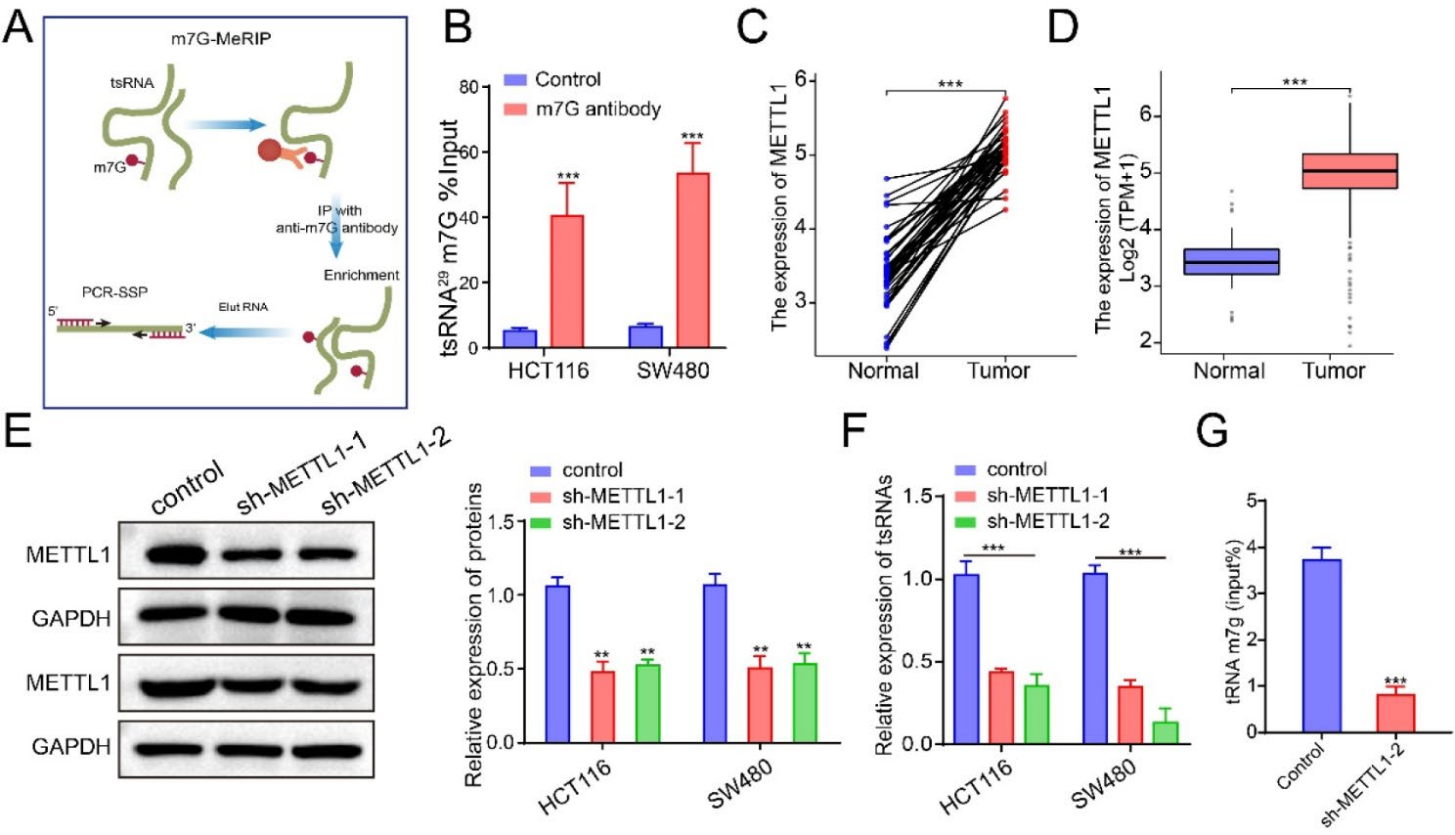
m^7^G modification on tsRNA-GlyGCC. **A**.The schematic diagram of m7G-MeRIP assay; **B**. m7G-MeRIP assay was performed to verification of m7G modification sites; **C**-**D**. The expression of METTL1 in CRC tissues; E. Interference efficiency of METTL1 by using sh-plasmid; **F**. tsRNA-GlyGCC was downexpressed in CRC cells transfected with sh-METTL1-1 and sh-METTL1-2; **G**. Interference with METTL1 expression could reduce the sequencing which containing site 29 enrichment. **p < 0.01, ***p < 0.001. All data are representative of at least three independent experiments and are presented as the means ± SD

m^7^G is one of the most common tRNA modifications in the tRNA variable loop, and is mediated by the METTL1-WDR4 complex [25]. Accumulating evidence has shown that m^7^G tRNA modifications are associated with a series of biological processes [42]. To explore whether tsRNA-GlyGCC undergoes m^7^G modification, we first predicted the m^7^G sites in 5’tRNA-Gly-GCC using a database (http://lin-group.cn/server/iRNA-m7G/predictor.php) and found several m^7^G sites (Figure S6A). Sites 29 and 45, which had the highest scores, was of particular interest, especially site 29, which formed from the tsRNA-GlyGCC sequence (Figure S6B-C). A schematic representation of the m^7^G-MeRIP assay is shown in Fig. 6A. As shown in Figure S6C, the sequencing of 5’tRNA-Gly-GCC and sequencing within site 45 were enriched in samples with the anti-7-methylguanosine antibody. Interestingly, sequences containing site 29 were significantly enriched in cells treated with anti-7-methylguanosine antibody (Fig. 6B). Additionally, the expression levels of METTL1 were upregulated in CRC according to the TCGA database (Fig. 6C-D). The sh-plasmid was used to interfere with METTL1 in CRC, and the results showed that sh-METTL1-1 and sh- METTL1-2 significantly reduced protein levels of METTL1 (Fig. 6E). Furthermore, the relative expression of tsRNA-GlyGCC decreased in CRC cells that reduced METTL1 (Fig. 6F), and sequencing containing site 29 was also decreased when reduce METTL1 expression (Fig. 6G). Additionally, we also detect the expression of other three tsRNA which was validated in Fig. 1 in METTL1-silenced cells. As showed in Figure S6D-F, the expression of tRF-28-Q99P9P9NH50E, tRF-32-J87383RPD9W1P, and tRF-27-OIQO4QPRJW3 did not show significant changes in METTL1-silenced cells compared with control. Overall, the upstream regulatory mechanism of tsRNA-GlyGCC involves m^7^G modification.

### In vitro synthesis and characterization of PAE@^5−FU^ts-inhibitor complex

**Fig. 7:**
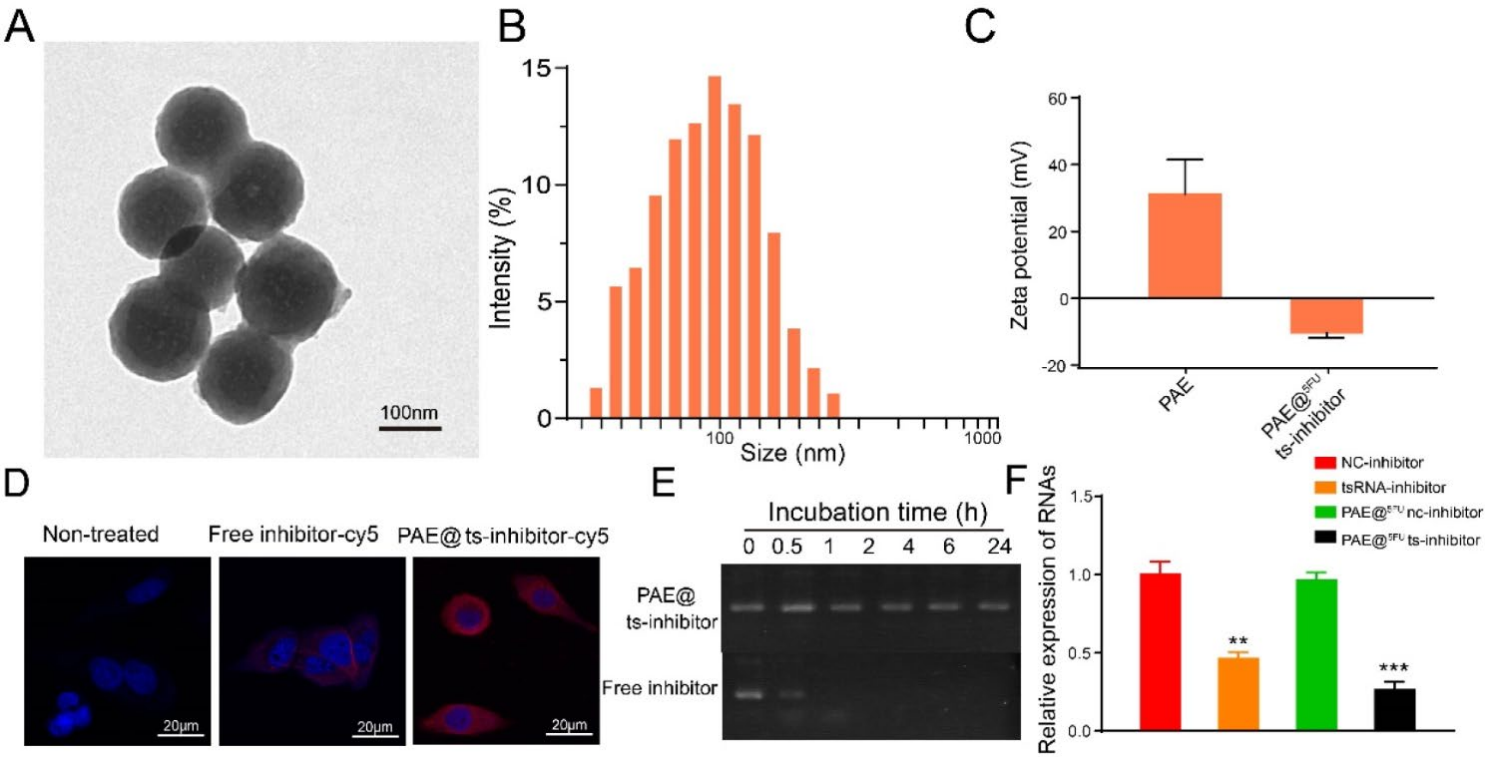
Characterization of PAE@^5−FU^ts-inhibitor complex. **A**.TEM images of PAE@^5−FU^ts-inhibitor complex; **B**. Particle size of the PAE@^5−FU^ts-inhibitor complex; **C**. Zeta potential of PAE and PAE@^5−FU^ts-inhibitor; **D**. Biodistribution of PAE@^5−FU^ts-inhibitor; **E**. Stability analysis of PAE@^5−FU^ts-inhibitor; **F**. The expression of tsRNA-GlyGCC.**p < 0.01, ***p < 0.001. All data are representative of at least three independent experiments and are presented as the means ± SD

Considering the notable role of tsRNA-GlyGCC in CRC, we sought to develop an efficient delivery system for tsRNA-GlyGCC-targeting inhibitors as a potential therapy for CRC. In this study, tsRNA-GlyGCC inhibitor-loaded nanocarriers were prepared as described in the Methods section. The morphology of the nanocarriers was characterized by transmission electron microscope (Fig. 7A), and the average particle size varied from 75 to 125 nm (Fig. 7B). The PAE carried positive charge, and after loading tsRNA-GlyGCC inhibitor, the zeta potential was reduced to -10.24 (Fig. 7C). Immunofluorescence staining showed that the tsRNA-GlyGCC inhibitor was mainly located in the cell cytoplasm, and the tsRNA-GlyGCC inhibitor-cy5 showed stronger fluorescence intensity when loaded in a nanoliposome of PAE, indicating that PAE has a stronger delivery efficiency (Fig. 7D). Furthermore, agarose gel electrophoresis indicated that PAE@ts-inhibitor shows more stability (Fig. 7E). The qPCR assay showed that the expression of tsRNA-GlyGCC was significantly downregulated in the PAE@^5−FU^ts-inhibitor groups compared to that in the tsRNA inhibitor group (Fig. 7F).

To investigate the antitumor effect of the PAE@^5−FU^ts-inhibitor in vitro, the CCK8 and clone formation assays was performed and it showed that the PAE@^5−FU^ts-inhibitor significantly reduced cell proliferation compared with the tsRNA-inhibitor groups (Figure S7A-B). In addition, transwell and flow cytometry assays indicated that the PAE@^5−FU^ts-inhibitor decreased cell migration ability and increased cell apoptosis compared with the tsRNA-inhibitor groups (Figure S7 C-D). These results indicate that the PAE@^5−FU^ts-inhibitor has an antitumor effect in vitro.

### The antitumor effects of PAE@^5−FU^ts-inhibitor in vivo

**Fig. 8:**
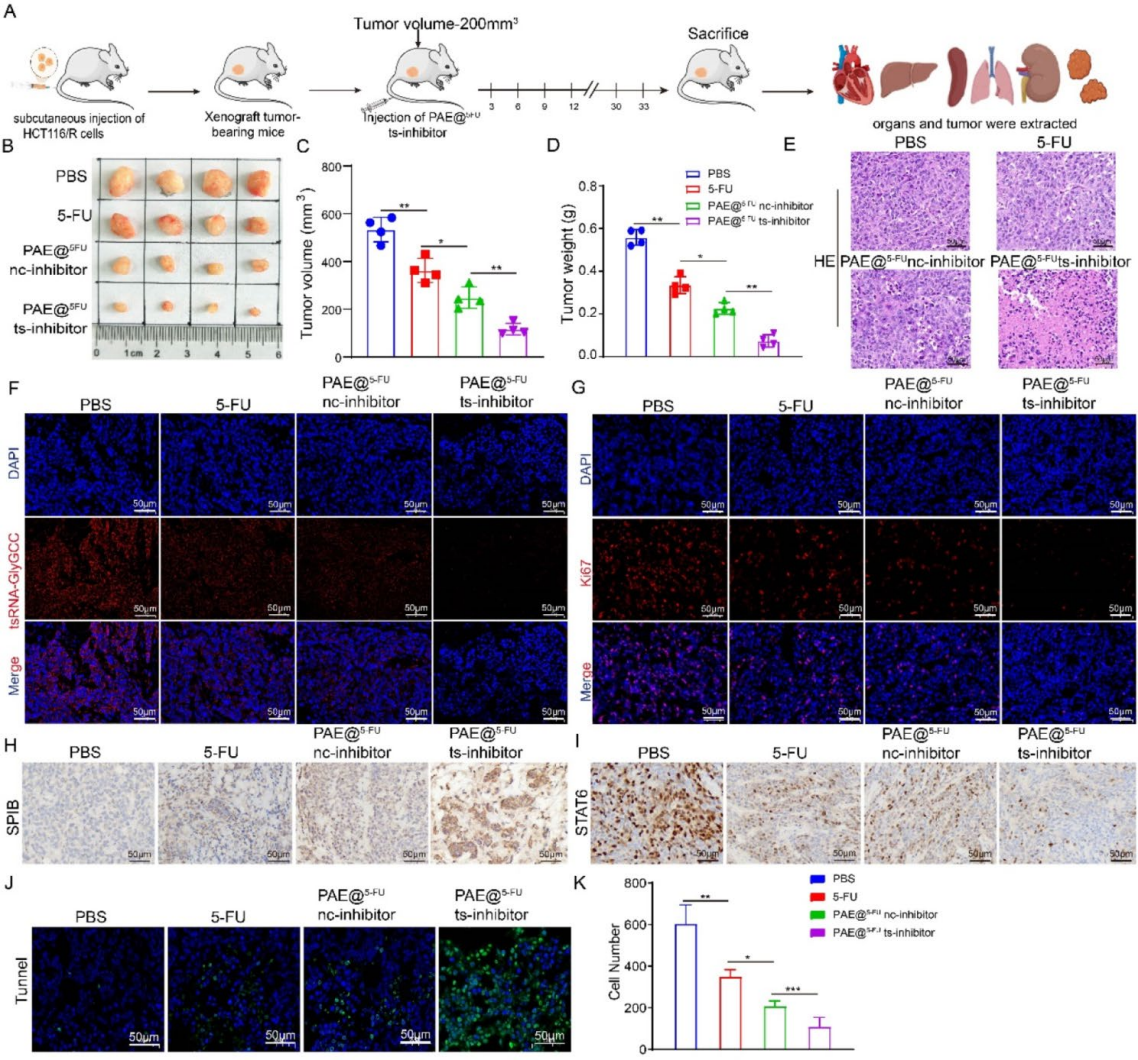
The antitumor effects of PAE@^5−FU^ts-inhibitor in vivo. **A**.The schematic diagram of mice treatment; **B**. Representative photos of tumours; **C**-**D**. The tumor volume and weight in different groups; **E**. HE staining of tumour cells in different groups; **F**. FISH assay was conducted to detect the expression of tsRNA-GlyGCC in different groups; G. Immunofluorescence staining was used to detect Ki67 expression in different groups; **H**-**I**. IHC was used to detected the expression of SPIB (H) and STAT6 (I) in tumor tissues; **J**-**K**.Tunnel assay was used to apoptosis in different groups; **p < 0.01, ***p < 0.001. All data are representative of at least three independent experiments and are presented as the means ± SD

The potential antitumor effects of PAE@^5−FU^ts-inhibitor in vivo. First, we evaluated the safety of PAE@^5−FU^ts-inhibitor in vivo. After treatment with the PBS, and PAE@^5−FU^ts-inhibitors, there were no significant changes in ALT, AST, BUN, or CREA (figure S8A). In addition, histological images of the liver, spleen, lungs, and kidneys showed no differences (figure S8B). An ideal delivery system for anticancer drugs should be able to better target tumor tissues. The inhibitors were labelled with Cy5 and loaded onto the PAE. The biological distribution was measured using in vivo imaging technology. The results shown in Figure S9A suggested that the fluorescence intensity of the tumor in PAE@ts-inhibitor-Cy5 was higher than that in tsRNA-inhibitor-Cy5. The uptake of PAE@ts-inhibitor-Cy5 was significantly higher than that of tsRNA-inhibitor-Cy5, and the red fluorescence in mice treated with PAE@ts-inhibitor-Cy5 was more significant than that in mice treated with tsRNA-inhibitor-Cy5 (Figure S9B-C). However, there were no significant differences in body weights between the two groups (Figure S9D). Subsequently, 5 × 10^5^ HCT116/R cells were transplanted subcutaneously into BALB/c mice. The experimental procedure is illustrated in Fig. 8A. The tumor size, volume, and weight of the PAE@^5−FU^ts-inhibitor were significantly lower than those of the PAE@^5−FU^nc-inhibitor (Fig. 8B-D). HE assays showed that the number of tumor cells in the PAE@^5−FU^ts-inhibitor groups had significantly reduced (Fig. 8E). FISH assay showed that tsRNA-GlyGCC was decreased in 5-FU treated tissues, and significantly decreased in tissues when treated with PAE@^5−FU^ts-inhibitor (Fig. 8F, Figure S9E). Immunofluorescence staining showed that the expression of the proliferation marker Ki67 decreased in the PAE@^5−FU^ts-inhibitor group (Fig. 8G, Figure S9F). Next, IHC assay was used to detect the expression of SPIB and STAT6 in tumor tissues. SPIB was significantly increased in tissues when treated with PAE@^5−FU^ts-inhibitor (Fig. 8H and Figure S9G), while STAT6 was decreased in tissues with PAE@^5−FU^ts-inhibitor treatment (Fig. 8I and Figure S9H). As showed in Fig. 8J-K, the number of apoptotic cells significantly increased, when treated with the PAE@^5−FU^ts-inhibitor. In all, PAE@^5−FU^ts-inhibitor showed higher anti-tumor effects.

### Antitumor effects of PAE@^5−FU^ts-inhibitor lung metastasis of colorectal cancer

To investigate the effect of the PAE@^5−FU^ts-inhibitor on lung metastasis, MC38-LUC cells were injected via the tail vein, followed by treatment with PBS, 5-FU, PAE@^5−FU^nc-inhibitor, and PAE@^5−FU^ts-inhibitor. The image of the lung tissue is showed in Figure S10A. The results indicated that the PAE@^5−FU^ts-inhibitor significantly reduced pulmonary metastatic nodules as well as the accumulation of 18^F^-FDG in the liver region (Figure S10B). The body weights of the mice showed no significant differences among the four groups (Figure S10C). The HE results were consistent with this result (Figure S10D). An IHC assay was also performed to examine Ki67 expression in lung tissues, and the results indicated that the protein levels of Ki67 were lower when treated with the PAE@^5−FU^ts-inhibitor (Figure S10E).

## Discussion

tsRNAs are a recently emerging group of sncRNAs that have been reported to participate in cancer development, cancer therapy, and drug resistance and may serve as tumor diagnostic/prognostic markers [43]. Based on the cellular dynamics of tsRNA research, it is speculated that they regulate protein-coding genes in an epigenetic way using RNA interference (RNAi) in a manner similar to miRNAs [44]. High-throughput genomic research has identified a large number of tsRNAs in CRC [45, 46]. One of these studies identified a novel 5’tiRNA-His-GTG, which showed involvement in signaling pathways that are involved in CRC progression. Hence, it may serve as a promising therapeutic target in CRC [19].

In the current study, we conducted tsRNA expression profiling in CRC tissues and screened four tsRNAs for expression level verification using qPCR. We identified a tsRNA-GlyGCC, tRF-31-P4R8YP9LON4VD, which was highly expressed in CRC tissues and cells. The correlation analysis between the expression of tsRNA-GlyGCC and clinical pathological features indicates that tsRNA-GlyGCC is positively correlated with tumor metastasis. As generally known, tumor metastasis was closely associated with cancer stem cells. Next, the in vitro experiments revealed that the tsRNA-GlyGCC inhibitor suppresses cell proliferation, migration, and formation of tumor spheres by modulating the protein expression of BCL2, BAX and cancer stem cells molecular markers (CD44,CD133, EphB2, LICAM, and LGR5). Through analysis of the enrichment of signaling pathways in target genes of tsRNA-GlyGCC. JAK-STAT signaling and drug resistance have attracted considerable attention. Several studies have reported that several cancer stem cells in cancer tissues contribute to chemo/radiotherapeutic resistance and metastasis, resulting in recurrence and death in patients [47]. The results of this study may help better understand the promotional effect of tsRNA-GlyGCC in CRC progression and drug resistance, since in vitro and in vivo experiments showed that inhibition of tsRNA-GlyGCC decreased the sensitivity of HCT116/R and SW480/R on 5-FU.

Although tsRNAs are involved in carcinogenesis by regulating transcription, altering mRNA stability, and inhibiting translation [48, 49], the biological mechanisms of tsRNAs in CRC drug resistance remain unclear. Similar to typical miRNA mechanism of mRNAs to silence target genes, tsRNA-GlyGCC was analyzed to target the 3’UTR of SPIB, which were experimentally verified by a luciferase report assay, western blotting, and qPCR. SPIB is an E-twenty-six transcription factor, which has been reported to be downregulated in CRC tissues, and overexpression of SPIB could decrease the IC50 values of oxaliplatin and 5-FU [41]. In our investigation, tsRNA-GlyGCC proved to negatively regulate SPIB and reduced the IC50 value of 5-FU in CRC 5-FU resistance cells. However, some studies have reported that SPIB plays a pro-tumor role, such as promotion of lung cancer invasion [50] and possible association with poor prognosis in patients with liver cancer [51]. For example, SPIB knockdown inhibits the immune escape of ovarian cancer cells and inactivates the JAK/STAT pathway. In this study, KEGG analysis shows that the target genes of tsRNA-GlyGCC were enriched in the JAK/STAT pathway. JAK/STAT pathway is a widely expressed intracellular signaling pathway that participates in many key biological processes, such as cell proliferation, apoptosis, differentiation, drug resistance and immune regulation [52]. Next, we detected the expression of STATs and JAKs in SPIB overexpressing cells. The results showed that overexpression of SPIB significantly inhibited STAT6. We speculated that SPIB regulate the expression of STAT6 in transcriptional regulatory. Using the JASPAR website, we identified two SPIB binding sites on the STAT6 promoter sequence with a score of over 9. The CHIP assay indicated that STAT6 could bind to the STAT6 promoter sequence at sites 266 to 15 and 1575 to 1351. Additionally, a rescue experiment was used to verify that JAK1/STAT6 pathway was involved in tsRNA-GlyGCC regulation of CRC carcinogenesis. All these results demonstrated that tsRNA-GlyGCC promote CRC 5-FU resistance by modulating the JAK1/STAT6 signaling pathway and by targeting SPIB.

RNA methylation occurs widely in eukaryotes and prokaryotes. Recently, m^7^G medication for tRNA has attracted considerable attention from researchers. METTL1-mediated tRNA modifications drive oncogenic transformation by increasing oncogenes proteins expression [53, 54]. We identified a predicted m7G site in tRNA-Gly-GCC using MeRIP and qPCR assays and found that METTL1 could bind to tRNA-Gly-GCC at sites 29 and 45. Our findings show that m^7^G modification of tRNA-Gly-GCC improves tsRNA-GlyGCC stability, which may partially account for the upregulation of tsRNA-GlyGCC in CRC.

The available therapeutic options for CRC remain restricted [55]. 5-FU has long been used as a standard first-line chemotherapeutic agent for CRC. However, resistance to 5-FU and dose-limiting cytotoxicity are the major factors limiting its anticancer efficacy in CRC [56, 57]. Therefore, in this study, we developed an inhibitor- and 5-FU-based NPs delivery system. PAEs are non-toxic and biodegradable materials that can be degraded by intracellular esterase, resulting in great improvement in biocompatibility, and hence proved to be an efficient delivery system for RNA and DNA [35, 58, 59]. In contrast, the PAE@^5−FU^ts-inhibitor complex increased inhibitor stability in serum and enhanced inhibitor uptake and accumulation by cancer cells, whereas PAE decreased the cytotoxicity of 5-FU and improved the delivery efficiency of 5-FU in cancer cells. To the best of our knowledge, this is the first study on the delivery of the tsRNA inhibitor and 5-FU PAEs in CRC. The PAE@^5−FU^ts-inhibitor complex significantly downregulated tsRNA-GlyGCC expression in CRC 5-FU resistance cells and exhibited effective antitumor effects in two animal models (subcutaneous mice and lung metastasis models). Notably, the PAE@^5−FU^ts-inhibitor showed no obvious toxicity in vitro, which suggested the feasibility and safety of the PAEs in delivering the tsRNA-GlyGCC inhibitor and 5-FU for drug-resistant cancer therapy. Further research should focus on the detailed regulatory mechanism of the PAE@^5FU^ts-inhibitor complex in drug resistance.

We confirmed the oncogenic role of tsRNA-GlyGCC and its molecular mechanism of action in 5-FU resistance in CRC both in vivo and in vitro. However, several limitations to our research need to be discussed. First, while we demonstrated the binding of tRNA-Gly-GCC to METTL1 in vitro, RNA pull-down should be used to further investigate the combination of tRNA-GlyGCC and METTL1, and the functional role of METTL1 should be studied. In addition, SPIB was confirmed to be a target of tsRNA-GlyGCC, and the anticancer role of SPIB in CRC drug resistance has been reported. Therefore, rescue experiments are needed to validate that tsRNA-GlyGCC promotes CRC 5-FU resistance by targeting SPIB. Third, the expression of SPIB was detected in the PAE@^5−FU^ts-inhibitor and control groups.

In conclusion, tsRNA-GlyGCC plays a tumorigenic role in CRC and promotes CRC 5-FU resistance by targeting SPIB and modulating the JAK1/STAT6 signaling pathway. Our study is the first to demonstrate the mechanism of tsRNA-GlyGCC in CRC 5-FU resistance and showed an effective approach to CRC therapy using the PAE@^5−FU^ts-inhibitor complex targeting tsRNA-GlyGCC, which provides a potential nanotherapeutic option for 5-FU sensitive and resistant CRC.

### Electronic supplementary material

Below is the link to the electronic supplementary material.

Supplementary Material 1

## Data Availability

The data in the current study are available from the corresponding author on reasonable request.

## References

[1] Sung H, Ferlay J, Siegel RL, Laversanne M, Soerjomataram I, Jemal A, Bray F. Global Cancer statistics 2020: GLOBOCAN estimates of incidence and Mortality Worldwide for 36 cancers in 185 countries. CA Cancer J Clin. 2021;71:209–49. 33538338 10.3322/caac.21660

[2] Di Y, Jing X, Hu K, Wen X, Ye L, Zhang X, Qin J, Ye J, Lin R, Wang Z, He W. The c-MYC-WDR43 signalling axis promotes chemoresistance and tumour growth in colorectal cancer by inhibiting p53 activity. Drug Resist Updat. 2023;66:100909. 36525936 10.1016/j.drup.2022.100909

[3] Jiang Z, Zou Q, Chen Q, Zhang J, Tang H, Chen J, Qin Y, Yang L, Chen Z, Cao L. Therapeutic role of Wuda granule in gastrointestinal motility disorder through promoting gastrointestinal motility and decreasing inflammatory level. Front Pharmacol. 2023;14:1237686. 37670946 10.3389/fphar.2023.1237686 PMC10476622

[4] Pita-Fernandez S, Alhayek-Ai M, Gonzalez-Martin C, Lopez-Calvino B, Seoane-Pillado T, Pertega-Diaz S. Intensive follow-up strategies improve outcomes in nonmetastatic colorectal cancer patients after curative surgery: a systematic review and meta-analysis. Ann Oncol. 2015;26:644–56. 25411419 10.1093/annonc/mdu543

[5] Xu Y, Liu K, Li C, Li M, Liu F, Zhou X, Sun M, Ranganathan M, Zhang L, Wang S, Hu X, Xu Y. The largest Chinese cohort study indicates homologous recombination pathway gene mutations as another major genetic risk factor for colorectal Cancer with heterogeneous clinical phenotypes. Research (Wash D C) 2023; 6: 0249. 10.34133/research.0249 PMC10581333 37854294

[6] Yang Y, Zhang J, Zhang W, Wang Y, Zhai Y, Li Y, Li W, Chang J, Zhao X, Huang M, Geng Q, Yang Y, Gong Z, Yu N, Shen W, Li Q, Huang S, Guo W. A liquid biopsy signature of circulating extracellular vesicles-derived RNAs predicts response to first line chemotherapy in patients with metastatic colorectal cancer. Mol Cancer. 2023;22:199. 38062470 10.1186/s12943-023-01875-y PMC10701920

[7] Wu Y, Song Y, Wang R, Wang T. Molecular mechanisms of tumor resistance to radiotherapy. Mol Cancer. 2023;22:96. 37322433 10.1186/s12943-023-01801-2 PMC10268375

[8] Yang C, Song J, Hwang S, Choi J, Song G, Lim W. Apigenin enhances apoptosis induction by 5-fluorouracil through regulation of thymidylate synthase in colorectal cancer cells. Redox Biol. 2021;47:102144. 34562873 10.1016/j.redox.2021.102144 PMC8476449

[9] McQuade RM, Stojanovska V, Bornstein JC, Nurgali K. Colorectal Cancer chemotherapy: the evolution of treatment and New approaches. Curr Med Chem. 2017;24:1537–57. 28079003 10.2174/0929867324666170111152436

[10] Del Rio M, Molina F, Bascoul-Mollevi C, Copois V, Bibeau F, Chalbos P, Bareil C, Kramar A, Salvetat N, Fraslon C, Conseiller E, Granci V, Leblanc B, Pau B, Martineau P, Ychou M. Gene expression signature in advanced colorectal cancer patients select drugs and response for the use of leucovorin, fluorouracil, and irinotecan. J Clin Oncol. 2007;25:773–80. 17327601 10.1200/JCO.2006.07.4187 PMC2257989

[11] Li X, Ma Y, Wu J, Ni M, Chen A, Zhou Y, Dai W, Chen Z, Jiang R, Ling Y, Yao Q, Chen W. Thiol oxidative stress-dependent degradation of transglutaminase2 via protein S-glutathionylation sensitizes 5-fluorouracil therapy in 5-fluorouracil-resistant colorectal cancer cells. Drug Resist Updat. 2023;67:100930. 36736043 10.1016/j.drup.2023.100930

[12] Chen L, Sun K, Qin W, Huang B, Wu C, Chen J, Lai Q, Wang X, Zhou R, Li A, Liu S, Zhang Y. LIMK1 m(6)A-RNA methylation recognized by YTHDC2 induces 5-FU chemoresistance in colorectal cancer via endoplasmic reticulum stress and stress granule formation. Cancer Lett. 2023;576:216420. 37778684 10.1016/j.canlet.2023.216420

[13] Deng X, Liao T, Xie J, Kang D, He Y, Sun Y, Wang Z, Jiang Y, Miao X, Yan Y, Tang H, Zhu L, Zou Y, Liu P. The burgeoning importance of PIWI-interacting RNAs in cancer progression. Sci China Life Sci. 2024;67:653–62. 38198029 10.1007/s11427-023-2491-7

[14] Ghasemian A, Omear HA, Mansoori Y, Mansouri P, Deng X, Darbeheshti F, Zarenezhad E, Kohansal M, Pezeshki B, Wang Z, Tang H. Long non-coding RNAs and JAK/STAT signaling pathway regulation in colorectal cancer development. Front Genet. 2023;14:1297093. 38094755 10.3389/fgene.2023.1297093 PMC10716712

[15] Anderson P, Ivanov P. tRNA fragments in human health and disease. FEBS Lett. 2014;588:4297–304. 25220675 10.1016/j.febslet.2014.09.001 PMC4339185

[16] Zhu L, Liu X, Pu W, Peng Y. tRNA-derived small non-coding RNAs in human disease. Cancer Lett. 2018;419:1–7. 29337107 10.1016/j.canlet.2018.01.015

[17] Li S, Xu Z, Sheng J, tRNA-Derived Small RNA. A Novel Regulatory Small non-coding RNA. Genes (Basel) 2018; 9. 10.3390/genes9050246 PMC5977186 29748504

[18] Li K, Lin Y, Luo Y, Xiong X, Wang L, Durante K, Li J, Zhou F, Guo Y, Chen S, Chen Y, Zhang D, Yeung SJ, Zhang H. A signature of saliva-derived exosomal small RNAs as predicting biomarker for esophageal carcinoma: a multicenter prospective study. Mol Cancer. 2022;21:21. 35042519 10.1186/s12943-022-01499-8 PMC8764835

[19] Tao EW, Wang HL, Cheng WY, Liu QQ, Chen YX, Gao QY. A specific tRNA half, 5’tiRNA-His-GTG, responds to hypoxia via the HIF1alpha/ANG axis and promotes colorectal cancer progression by regulating LATS2. J Exp Clin Cancer Res. 2021;40:67. 33588913 10.1186/s13046-021-01836-7 PMC7885485

[20] Goodarzi H, Liu X, Nguyen HC, Zhang S, Fish L, Tavazoie SF. Endogenous tRNA-Derived fragments suppress breast Cancer progression via YBX1 displacement. Cell. 2015;161:790–802. 25957686 10.1016/j.cell.2015.02.053 PMC4457382

[21] Tomikawa C. 7-Methylguanosine modifications in transfer RNA (tRNA). Int J Mol Sci 2018; 19. 10.3390/ijms19124080 PMC6320965 30562954

[22] Zhao Y, Kong L, Pei Z, Li F, Li C, Sun X, Shi B, Ge J. m7G methyltransferase METTL1 promotes post-ischemic angiogenesis via promoting VEGFA mRNA translation. Front Cell Dev Biol. 2021;9:642080. 34136476 10.3389/fcell.2021.642080 PMC8200671

[23] Luo Y, Yao Y, Wu P, Zi X, Sun N, He J. The potential role of N(7)-methylguanosine (m7G) in cancer. J Hematol Oncol. 2022;15:63. 35590385 10.1186/s13045-022-01285-5 PMC9118743

[24] Huang M, Long J, Yao Z, Zhao Y, Zhao Y, Liao J, Lei K, Xiao H, Dai Z, Peng S, Lin S, Xu L, Kuang M. METTL1-Mediated m7G tRNA modification promotes Lenvatinib Resistance in Hepatocellular Carcinoma. Cancer Res. 2023;83:89–102. 36102722 10.1158/0008-5472.CAN-22-0963

[25] Han H, Yang C, Ma J, Zhang S, Zheng S, Ling R, Sun K, Guo S, Huang B, Liang Y, Wang L, Chen S, Wang Z, Wei W, Huang Y, Peng H, Jiang YZ, Choe J, Lin S. N(7)-methylguanosine tRNA modification promotes esophageal squamous cell carcinoma tumorigenesis via the RPTOR/ULK1/autophagy axis. Nat Commun. 2022;13:1478. 35304469 10.1038/s41467-022-29125-7 PMC8933395

[26] Jakab G, Kis M, Palfi Z, Solymosy F. Nucleotide sequence of chloroplast tRNA(Leu)/UA m7G/from Chlamydomonas reinhardtii. Nucleic Acids Res. 1990;18:7444. 2259637 10.1093/nar/18.24.7444 PMC332888

[27] Matsuyama S, Ueda T, Crain PF, McCloskey JA, Watanabe K. A novel wobble rule found in starfish mitochondria. Presence of 7-methylguanosine at the Anticodon wobble position expands decoding capability of tRNA. J Biol Chem. 1998;273:3363–8. 9452455 10.1074/jbc.273.6.3363

[28] Luo M, Yang X, Chen HN, Nice EC, Huang C. Drug resistance in colorectal cancer: an epigenetic overview. Biochim Biophys Acta Rev Cancer. 2021;1876:188623. 34481016 10.1016/j.bbcan.2021.188623

[29] Zhang Y, Qian H, He J, Gao W. Mechanisms of tRNA-derived fragments and tRNA halves in cancer treatment resistance. Biomark Res. 2020;8:52. 33072328 10.1186/s40364-020-00233-0 PMC7559774

[30] Primeaux M, Liu X, Gowrikumar S, Fatima I, Fisher KW, Bastola D, Vecchio AJ, Singh AB, Dhawan P. Claudin-1 interacts with EPHA2 to promote cancer stemness and chemoresistance in colorectal cancer. Cancer Lett. 2023;579:216479. 37924938 10.1016/j.canlet.2023.216479 PMC10765961

[31] Fu BF, Xu CY. Transfer RNA-Derived small RNAs: novel regulators and biomarkers of cancers. Front Oncol. 2022;12:843598. 35574338 10.3389/fonc.2022.843598 PMC9096126

[32] Cui Y, Huang Y, Wu X, Zheng M, Xia Y, Fu Z, Ge H, Wang S, Xie H. Hypoxia-induced tRNA-derived fragments, novel regulatory factor for doxorubicin resistance in triple-negative breast cancer. J Cell Physiol. 2019;234:8740–51. 30362543 10.1002/jcp.27533

[33] Sun C, Yang F, Zhang Y, Chu J, Wang J, Wang Y, Zhang Y, Li J, Li Y, Fan R, Li W, Huang X, Wu H, Fu Z, Jiang Z, Yin Y. tRNA-Derived fragments as novel predictive biomarkers for Trastuzumab-resistant breast Cancer. Cell Physiol Biochem. 2018;49:419–31. 30153663 10.1159/000492977

[34] Raoof M, Whelan RL, Sullivan KM, Ruel C, Frankel PH, Cole SE, Tinsley R, Eng M, Fakih M, Chao J, Lim D, Woo Y, Paz IB, Lew M, Cristea M, Rodriguez-Rodriguez L, Fong Y, Thomas RM, Chang S, Deperalta D, Merchea A, Dellinger TH. Safety and Efficacy of Oxaliplatin pressurized Intraperitoneal Aerosolized Chemotherapy (PIPAC) in colorectal and Appendiceal Cancer with peritoneal metastases: results of a Multicenter Phase I Trial in the USA. Ann Surg Oncol. 2023;30:7814–24. 37501051 10.1245/s10434-023-13941-2 PMC10562297

[35] Du A, Li S, Zhou Y, Disoma C, Liao Y, Zhang Y, Chen Z, Yang Q, Liu P, Liu S, Dong Z, Razzaq A, Tao S, Chen X, Liu Y, Xu L, Zhang Q, Li S, Peng J, Xia Z. M6A-mediated upregulation of circMDK promotes tumorigenesis and acts as a nanotherapeutic target in hepatocellular carcinoma. Mol Cancer. 2022;21:109. 35524319 10.1186/s12943-022-01575-z PMC9074191

[36] Wu P, Zhou Q, Zhu H, Zhuang Y, Bao J. Enhanced antitumor efficacy in colon cancer using EGF functionalized PLGA nanoparticles loaded with 5-Fluorouracil and perfluorocarbon. BMC Cancer. 2020;20:354. 32345258 10.1186/s12885-020-06803-7 PMC7189558

[37] Dong S, Liang S, Cheng Z, Zhang X, Luo L, Li L, Zhang W, Li S, Xu Q, Zhong M, Zhu J, Zhang G, Hu. ROS/PI3K/Akt and Wnt/beta-catenin signalings activate HIF-1alpha-induced metabolic reprogramming to impart 5-fluorouracil resistance in colorectal cancer. J Exp Clin Cancer Res. 2022;41:15. 34998404 10.1186/s13046-021-02229-6 PMC8742403

[38] Xu R, Yang Q. Immunological significance of prognostic markers for breast cancer based on alternative splicing. Am J Transl Res. 2022;14:4229–50. 35836866 PMC9274553

[39] Liang J, Dai W, Liu C, Wen Y, Chen C, Xu Y, Huang S, Hou S, Li C, Chen Y, Wang W, Tang H. Gingerenone A attenuates Ulcerative Colitis via Targeting IL-17RA to inhibit inflammation and restore intestinal barrier function. Adv Sci (Weinh) 2024; e2400206. 10.1002/advs.202400206 PMC11267284 38639442

[40] Mishra P, Singh U, Pandey CM, Mishra P, Pandey G. Application of student’s t-test, analysis of variance, and covariance. Ann Card Anaesth. 2019;22:407–11. 31621677 10.4103/aca.ACA_94_19 PMC6813708

[41] Zhao X, Li L, Yuan S, Zhang Q, Jiang X, Luo T. SPIB acts as a tumor suppressor by activating the NFkB and JNK signaling pathways through MAP4K1 in colorectal cancer cells. Cell Signal. 2021;88:110148. 34530056 10.1016/j.cellsig.2021.110148

[42] Dai Z, Liu H, Liao J, Huang C, Ren X, Zhu W, Zhu S, Peng B, Li S, Lai J, Liang L, Xu L, Peng S, Lin S, Kuang M. N(7)-Methylguanosine tRNA modification enhances oncogenic mRNA translation and promotes intrahepatic cholangiocarcinoma progression. Mol Cell. 2021;81:3339–e33553338. 34352206 10.1016/j.molcel.2021.07.003

[43] Peterman TA, Lui KJ, Lawrence DN, Allen JR. Estimating the risks of transfusion-associated acquired immune deficiency syndrome and human immunodeficiency virus infection. Transfusion. 1987;27:371–4. 2888222 10.1046/j.1537-2995.1987.27587320525.x

[44] Haussecker D, Huang Y, Lau A, Parameswaran P, Fire AZ, Kay MA. Human tRNA-derived small RNAs in the global regulation of RNA silencing. RNA. 2010;16:673–95. 20181738 10.1261/rna.2000810 PMC2844617

[45] Xiong W, Wang X, Cai X, Xiong W, Liu Y, Li C, Liu Q, Qin J, Li Y. Identification of tRNA–derived fragments in colon cancer by comprehensive small RNA sequencing. Oncol Rep. 2019;42:735–44. 31173257 10.3892/or.2019.7178

[46] Wang XY, Zhou YJ, Chen HY, Chen JN, Chen SS, Chen HM, Li XB. 5’tiRNA-Pro-TGG, a novel tRNA halve, promotes oncogenesis in sessile serrated lesions and serrated pathway of colorectal cancer. World J Gastrointest Oncol. 2023;15:1005–18. 37389118 10.4251/wjgo.v15.i6.1005 PMC10302996

[47] Bai X, Ni J, Beretov J, Graham P, Li Y. Cancer stem cell in breast cancer therapeutic resistance. Cancer Treat Rev. 2018;69:152–63. 30029203 10.1016/j.ctrv.2018.07.004

[48] Yu X, Xie Y, Zhang S, Song X, Xiao B, Yan Z. tRNA-derived fragments: mechanisms underlying their regulation of gene expression and potential applications as therapeutic targets in cancers and virus infections. Theranostics. 2021;11:461–9. 33391486 10.7150/thno.51963 PMC7681095

[49] Xiao L, Wang J, Ju S, Cui M, Jing R. Disorders and roles of tsRNA, snoRNA, snRNA and piRNA in cancer. J Med Genet. 2022;59:623–31. 35145038 10.1136/jmedgenet-2021-108327

[50] Du W, Xu X, Niu Q, Zhang X, Wei Y, Wang Z, Zhang W, Yan J, Ru Y, Fu Z, Li X, Jiang Y, Ma Z, Zhang Z, Yao Z, Liu Z. Spi-B-Mediated silencing of Claudin-2 promotes early dissemination of Lung Cancer cells from primary tumors. Cancer Res. 2017;77:4809–22. 28754672 10.1158/0008-5472.CAN-17-0020

[51] Takagi Y, Shimada K, Shimada S, Sakamoto A, Naoe T, Nakamura S, Hayakawa F, Tomita A, Kiyoi H. SPIB is a novel prognostic factor in diffuse large B-cell lymphoma that mediates apoptosis via the PI3K-AKT pathway. Cancer Sci. 2016;107:1270–80. 27348272 10.1111/cas.13001 PMC5021043

[52] Xin P, Xu X, Deng C, Liu S, Wang Y, Zhou X, Ma H, Wei D, Sun S. The role of JAK/STAT signaling pathway and its inhibitors in diseases. Int Immunopharmacol. 2020;80:106210. 31972425 10.1016/j.intimp.2020.106210

[53] Orellana EA, Liu Q, Yankova E, Pirouz M, De Braekeleer E, Zhang W, Lim J, Aspris D, Sendinc E, Garyfallos DA, Gu M, Ali R, Gutierrez A, Mikutis S, Bernardes GJL, Fischer ES, Bradley A, Vassiliou GS, Slack FJ. Tzelepis K and Gregory RI. METTL1-mediated m(7)G modification of Arg-TCT tRNA drives oncogenic transformation. Mol Cell. 2021;81:3323–38. e3314. 34352207 10.1016/j.molcel.2021.06.031 PMC8380730

[54] Garcia-Vilchez R, Anazco-Guenkova AM, Dietmann S, Lopez J, Moron-Calvente V, D’Ambrosi S, Nombela P, Zamacola K, Mendizabal I, Garcia-Longarte S, Zabala-Letona A, Astobiza I, Fernandez S, Paniagua A, Miguel-Lopez B, Marchand V, Alonso-Lopez D, Merkel A, Garcia-Tunon I, Ugalde-Olano A, Loizaga-Iriarte A, Lacasa-Viscasillas I, Unda M, Azkargorta M, Elortza F, Barcena L, Gonzalez-Lopez M, Aransay AM, Di Domenico T, Sanchez-Martin MA, De Las Rivas J, Guil S, Motorin Y, Helm M, Pandolfi PP. Carracedo A and Blanco S. METTL1 promotes tumorigenesis through tRNA-derived fragment biogenesis in prostate cancer. Mol Cancer. 2023;22:119. 37516825 10.1186/s12943-023-01809-8 PMC10386714

[55] Guo W, Cai Y, Liu X, Ji Y, Zhang C, Wang L, Liao W, Liu Y, Cui N, Xiang J, Li Z, Wu D, Li J. Single-exosome profiling identifies ITGB3 + and ITGAM + exosome subpopulations as promising early diagnostic biomarkers and therapeutic targets for Colorectal Cancer. Res (Wash D C). 2023;6:0041. 10.34133/research.0041 PMC10076010 37040507

[56] Wei Y, Yang P, Cao S, Zhao L. The combination of curcumin and 5-fluorouracil in cancer therapy. Arch Pharm Res. 2018;41:1–13. 29230689 10.1007/s12272-017-0979-x

[57] Vodenkova S, Buchler T, Cervena K, Veskrnova V, Vodicka P, Vymetalkova V. 5-fluorouracil and other fluoropyrimidines in colorectal cancer: past, present and future. Pharmacol Ther. 2020;206:107447. 31756363 10.1016/j.pharmthera.2019.107447

[58] Yin Q, Gao Y, Zhang Z, Zhang P, Li Y. Bioreducible poly (beta-amino esters)/shRNA complex nanoparticles for efficient RNA delivery. J Control Release. 2011;151:35–44. 21244853 10.1016/j.jconrel.2010.12.014

[59] Shi J, Zhang Y, Ma B, Yong H, Che D, Pan C, He W, Zhou D, Li M. Enhancing the gene transfection of poly(beta-amino ester)/DNA polyplexes by modular manipulation of Amphiphilicity. ACS Appl Mater Interfaces. 2023;15:42130–8. 37642943 10.1021/acsami.3c03802

