# Supplementary material for: tsRNA-GlyGCC promotes colorectal cancer progression and 5-FU resistance by regulating SPIB
Source: J Exp Clin Cancer Res. 2024 Aug 17;43:230. doi: 10.1186/s13046-024-03132-6 (PMC11330149; doi:10.1186/s13046-024-03132-6)
Supplement: Supplementary file 1 — Supplementary Material 1 [file 13046_2024_3132_MOESM1_ESM.docx]

Supplementary figures:


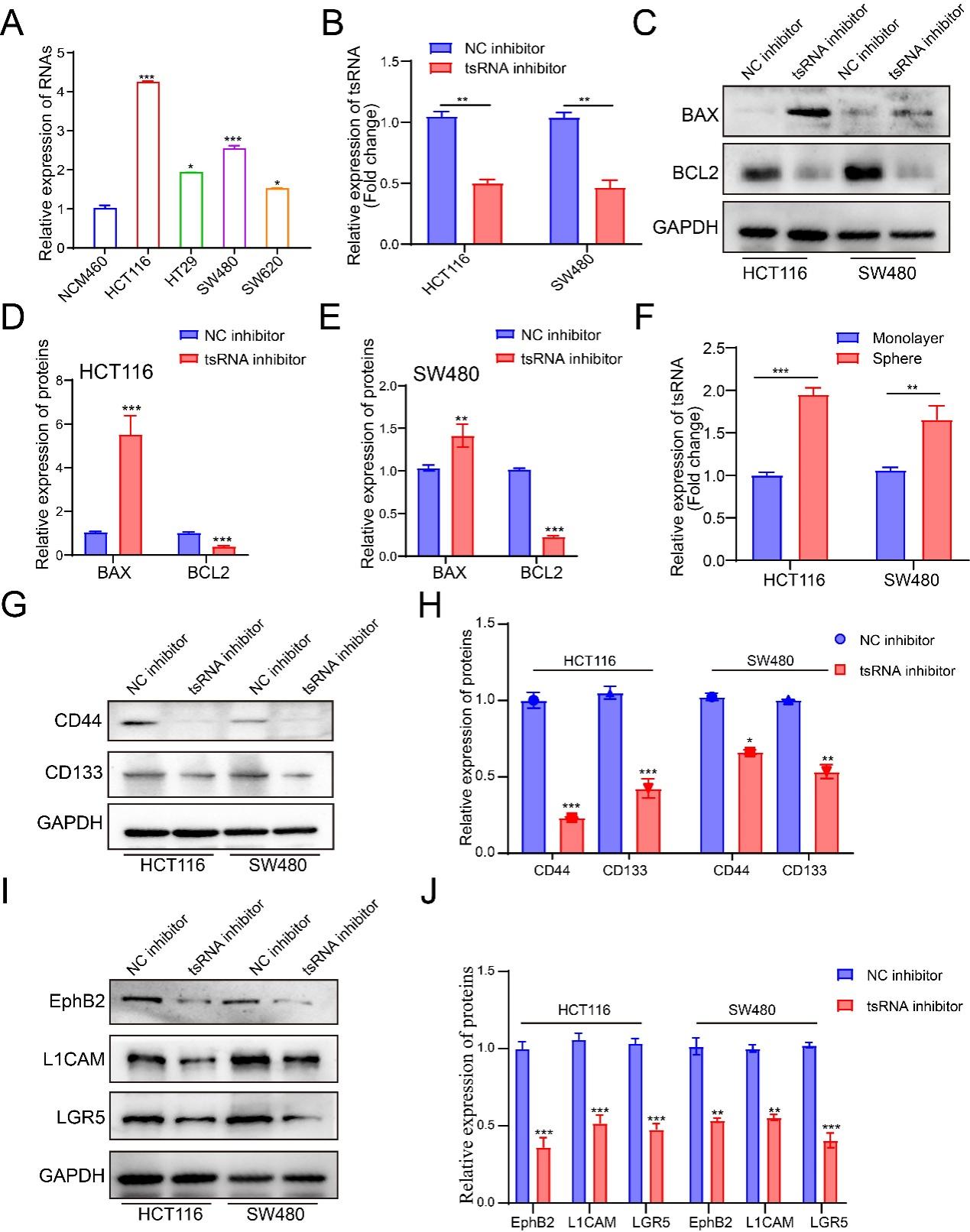


**Figure S1** The expression of tsRNA-GlyGCC in CRC cells and CRC sphere.

1. tsRNA-GlyGCC was increased in CRC cells compared with normal colonic

epithelial cells; B. tsRNA-GlyGCC was decreased in CRC cells transfected with specific tsRNA-GlyGCC inhibitor; C-E. tsRNA-GlyGCC inhibitor decreased the expression of BCL2, while increased the expression of BAX; F. tsRNA-GlyGCC was increased in CRC sphere; G-H. The protein level of CD44 and CD133 in NC inhibitor group and tsRNA-GlyGCC inhibitor group; I-J. The protein level of EphB2, LICAM, and LGR5 were downregulated in tsRNA-GlyGCC inhibitor group. *p<0.05, **p < 0.01, ***p < 0.001. All data are representative of at least three independent experiments and are presented as the means ± SD.


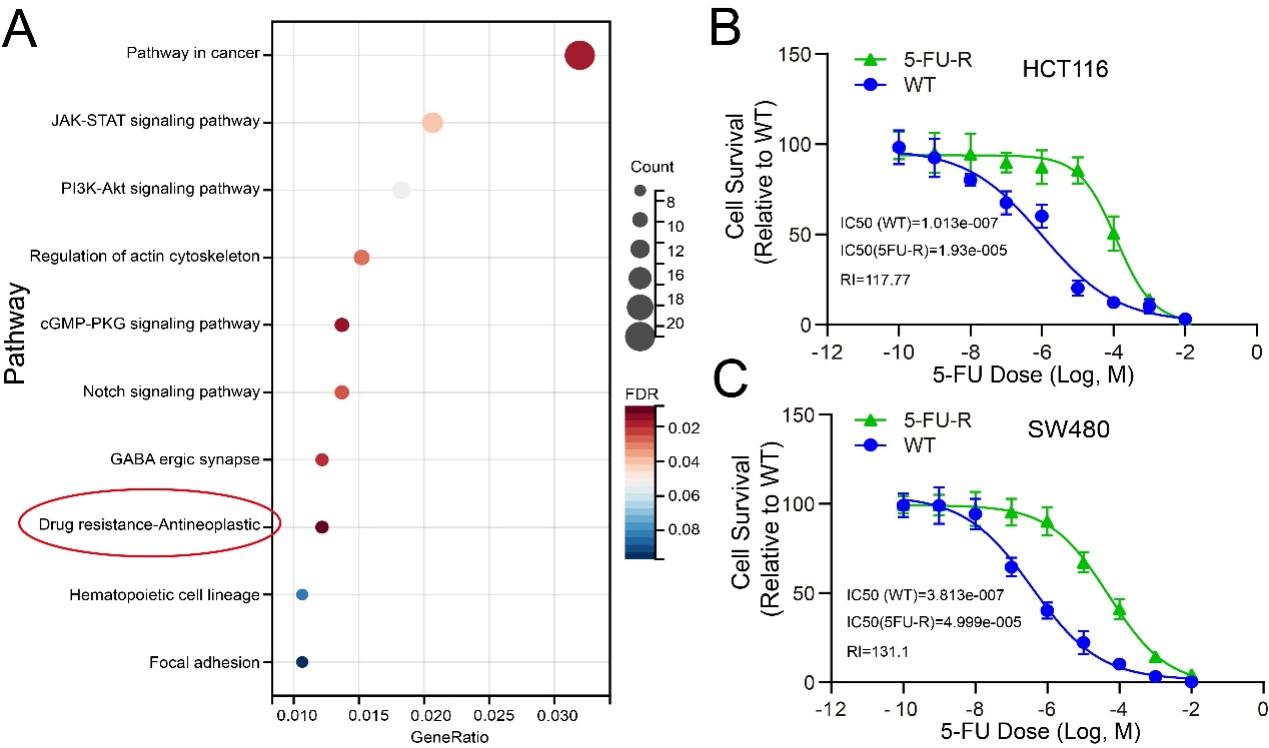


**Figure S2** establishment of the acquired 5-FU-R CRC cell lines

A. KEGG analysis the enrichment of signaling pathway in target genes of tsRNA-GlyGCC; B. CCK8 assays to assess 5-FU sensitivity of CRC cells.


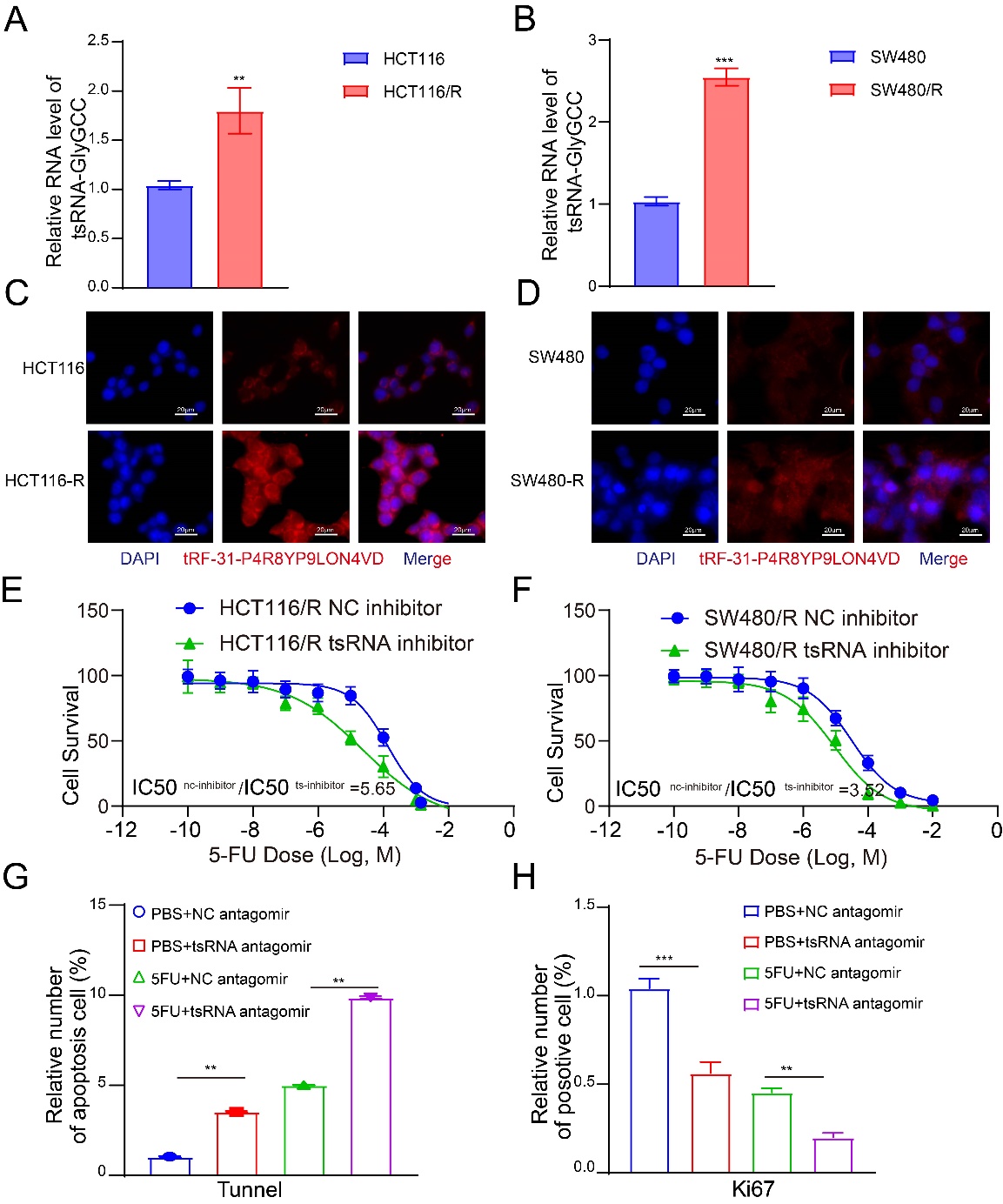


**Figure S3** tsRNA-GlyGCC inhibitor enhance sensitive of CRC 5-FU resistance cells to 5-FU

A-B. qPCR was used to detect the expression of tsRNA-GlyGCC in CRC cells and CRC resistance cells. C-D. FISH was used to detect the expression of tsRNA-GlyGCC in CRC cells and CRC resistance cells, merged images represent overlays of tsRNA-GlyGCC and nuclear stained with DAPI in blue. E-F. CCK8 was used to detect cell survival in HCT116 and SW480 resistance cells; G. Statistical analysis for tunnel assay; H. Statistical analysis for IF assay. **p < 0.01, ***p < 0.001. All data are representative of at least three independent experiments and are presented as the means ± SD.


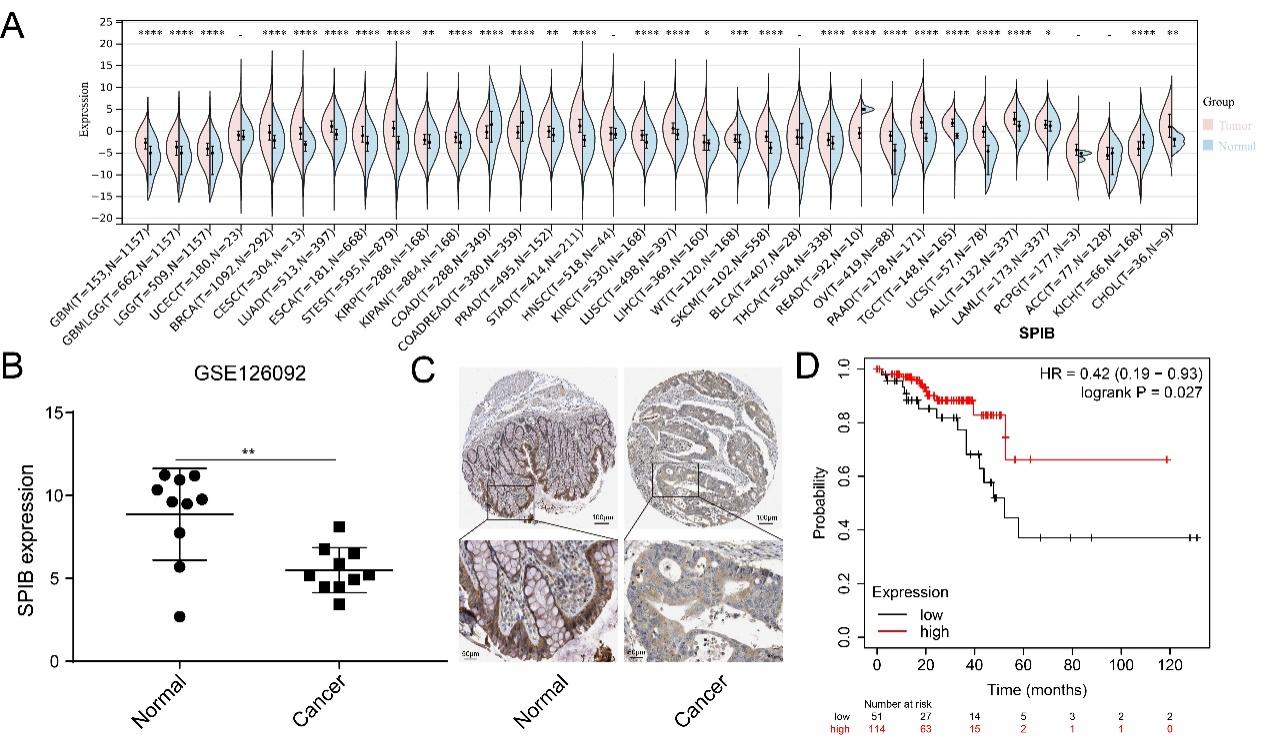


**Figure S4 The expression of SPIB in CRC tissues**

A.The expression of SPIB in pan-cancers; B. The expression of SPIB in GSE126092 dataset; C. Protein level of SPIB in CRC tissues; D. DFS curves of CRC patients were associated with SPIB expression. **p < 0.01.


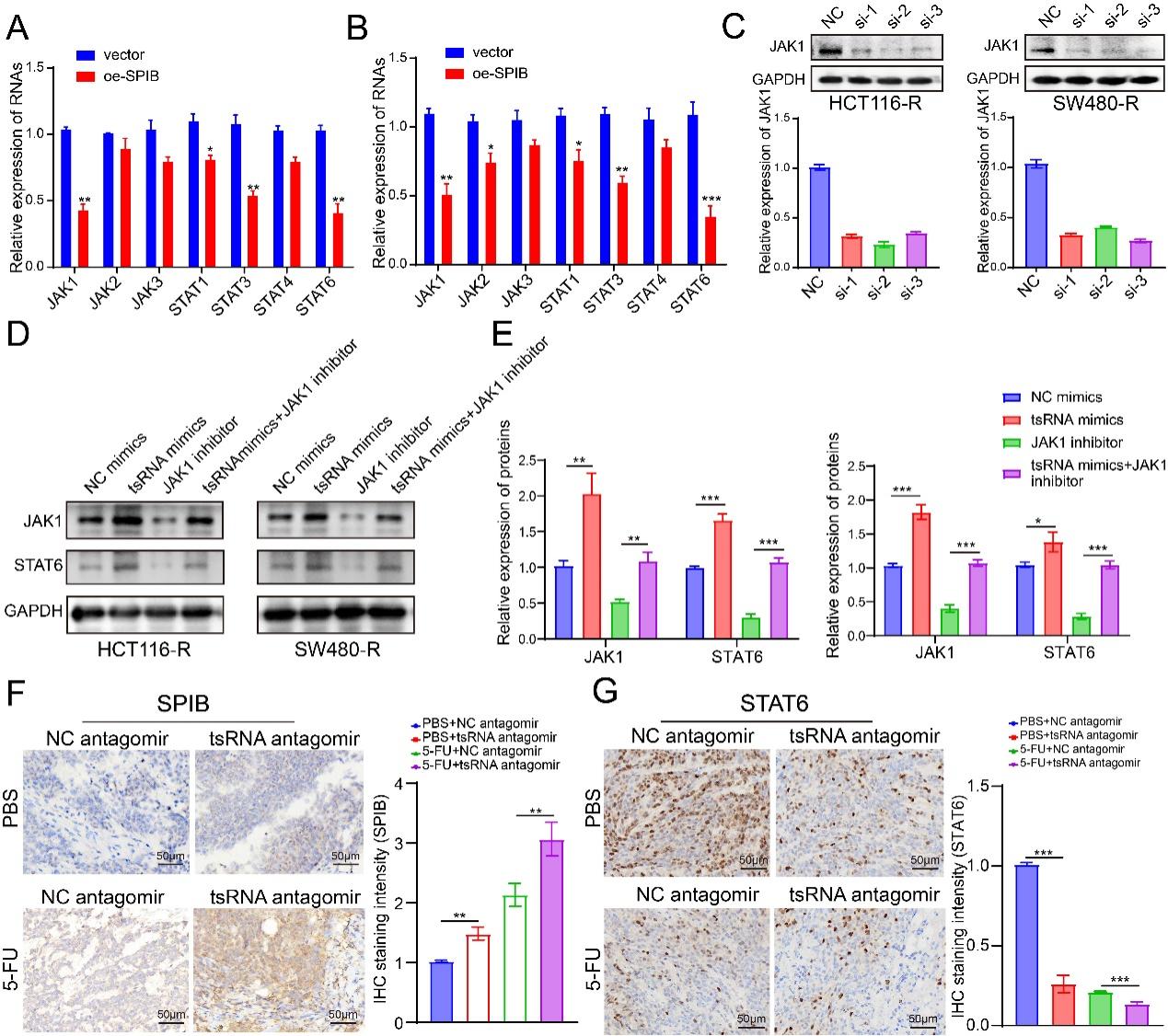


**Figure S5 SPIB regulating JAK/STAT6 signaling pathway.**

A-B. The mRNA level of JAK1/STAT6 were reduced in overexpression SPIB cells; C. qPCR was used to determine the expression of JAK1 after transfected with specific siRNA; D-E. WB was used to detect the protein level of JAK1 and STAT6 in different groups; F-G. IHC was used to detect the protein level of SPIB (F) and STAT6 (G) in tumor tissues from figure 3. *p<0.05, **p < 0.01, ***p < 0.001. All data are representative of at least three independent experiments and are presented as the means ± SD


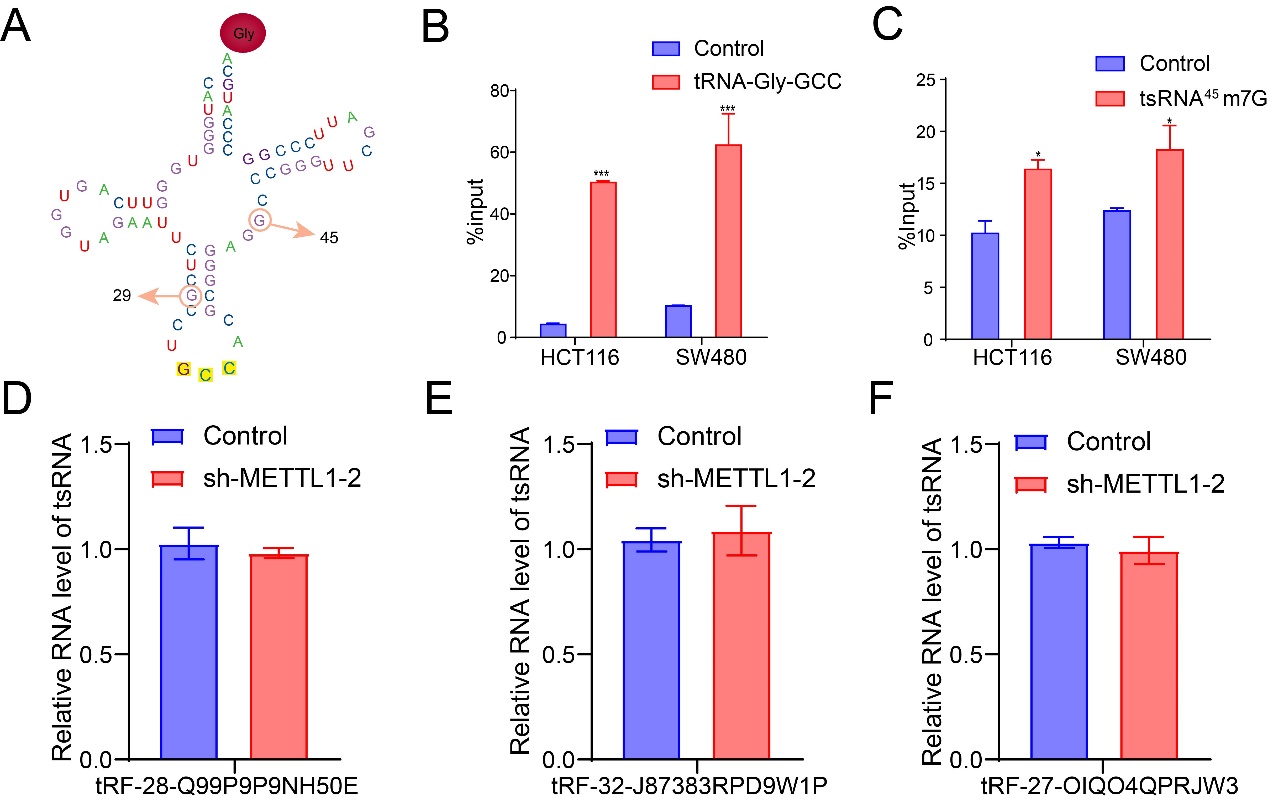


**Figure S6 m^7^G modification prediction**

A. Online tools was used to analysis the m^7^G modification sites; B-C. m^7^G-MeRIP assay was performed to test the m^7^G modification on tRNA-Gly-GCC (left) and tsRNA^45^ (right); D-F. qPCR was used to detect the expression of RF-30-J87383RPD9W1, tRF-32-J87383RPD9W1P, and tRF-27-OIQO4QPRJW3 in METTL1-silenced cells. *p<0.05, ***p < 0.001. All data are representative of at least three independent experiments and are presented as the means ± SD.


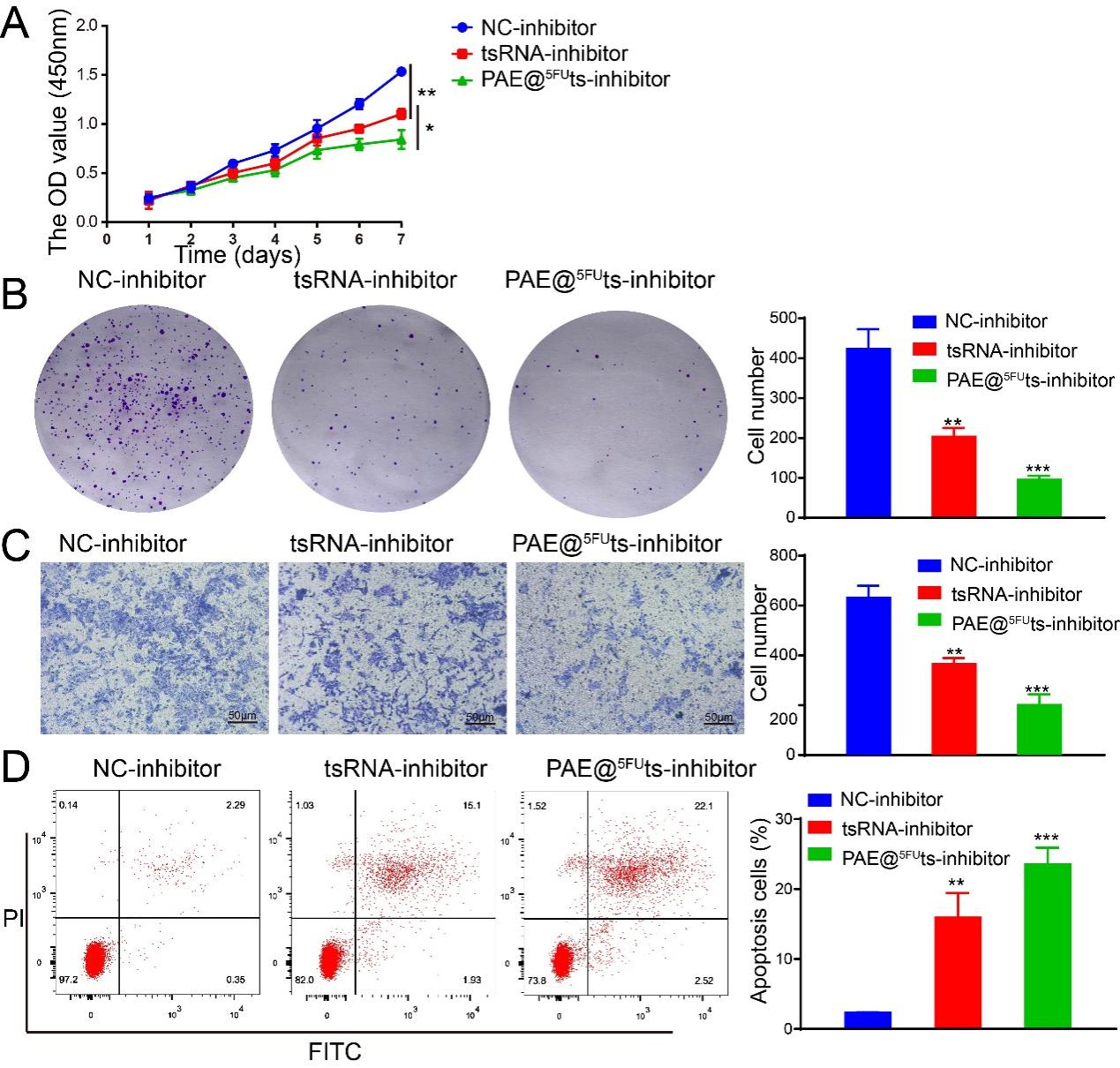


**Figure S7 the antitumor effect of PAE@^5-FU^ts-inhibitor in vitro**

A-B. CCk8 assay and colony formation assay was used to detect the cell proliferation; C. Transwell assay was used for migration ability assessment; D. Flow cytometry analysis showing apoptosis in different groups. *p<0.05, **p < 0.01, ***p < 0.001. All data are representative of at least three independent experiments and are presented as the means ± SD.


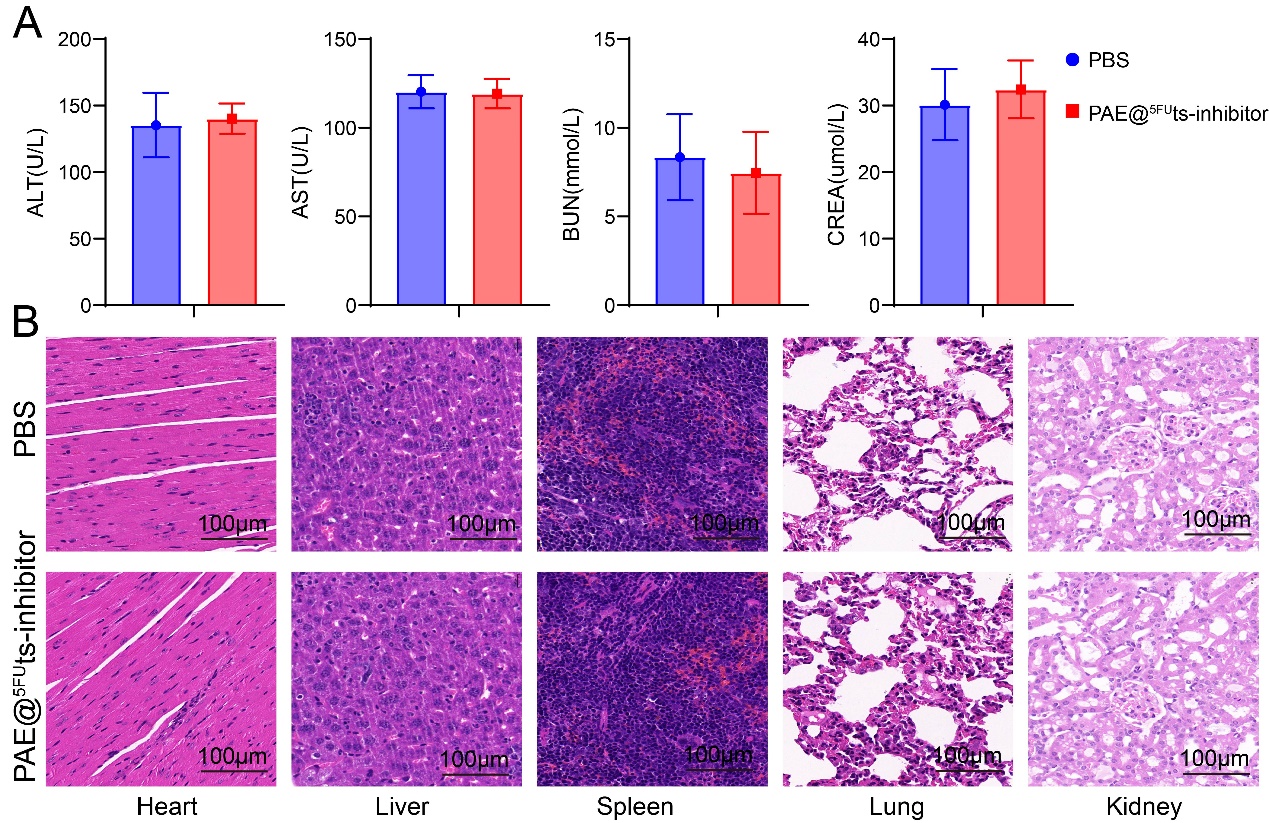


**Figure S8 biological safety of PAE@^5-FU^ts-inhibitor in vitro**

A.Changes of liver function and renal function index (ALT, AST, BUN and CREA); B.The histological images of liver, spleen, lung and kidney in different groups.


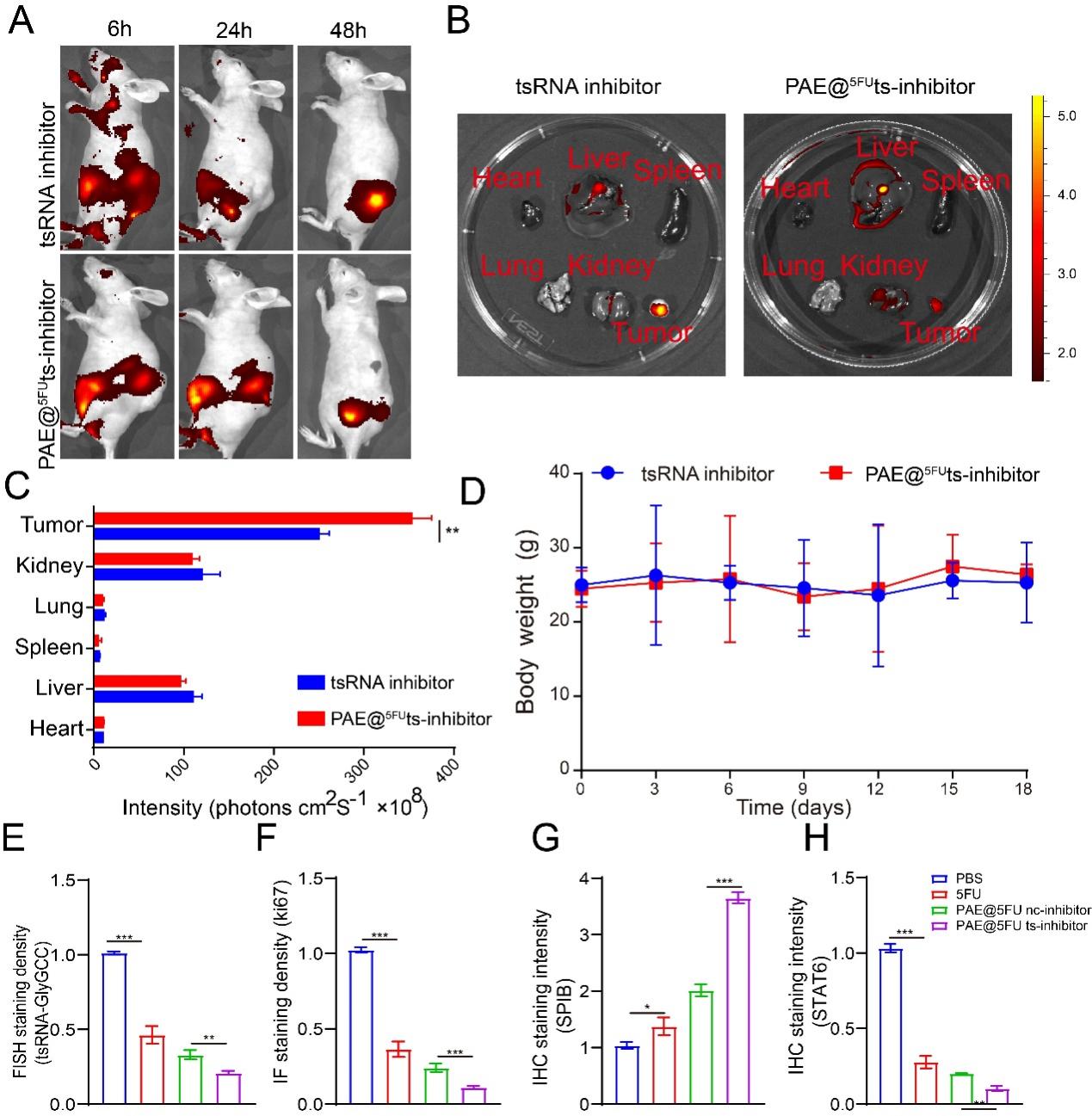


**Figure S9 Verification of PAE@^5-FU^ts-inhibitor targeting in balb/c nude mice**

A.The fluorescence images in vivo were observed at different point of time; B-C. analysis for ex vivo bioluminescent images of liver, spleen, lung and kidney and tumors at 48 h post injection; D. The body weight of mice in different groups; E. Statistical analysis for FISH assay.; F. Statistical analysis for IF assay; G-H. Statistical analysis for the protein level of SPIB (G) and STAT6 (H); *p<0.05, **p < 0.01, ***p < 0.001. All data are representative of at least three independent experiments and are presented as the means ± SD.


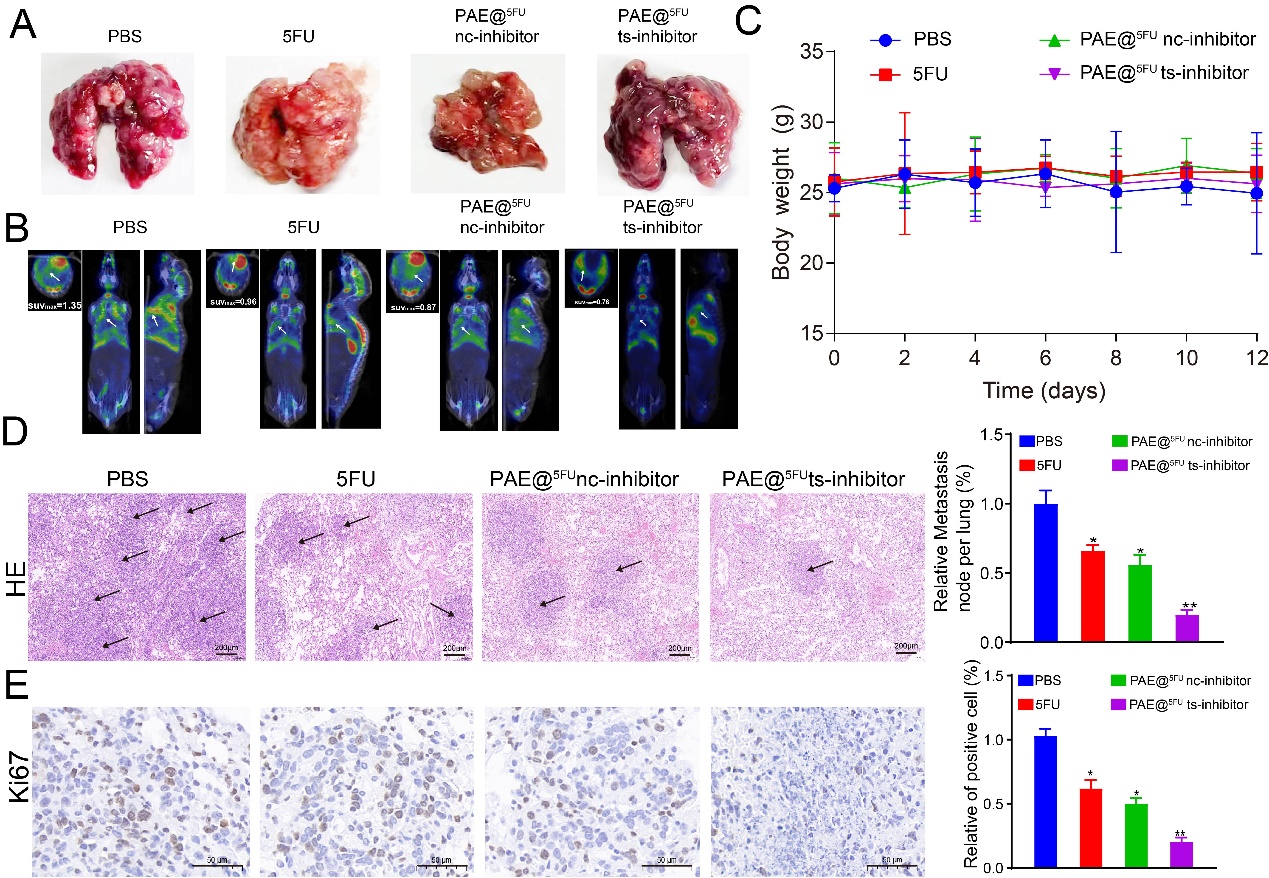


**Figure S10** **Antitumor effects of PAE@^5-FU^ts-inhibitor lung metastasis of colorectal cancer**

A.Representative images of the MC38-LUC lung metastasis of CRC; B. Representative images of PET/CT scans from each group; C. The weight body of mice in different groups; D. Representative images of liver sections stained with HE; E. Representative images of IHC stained with ki67. *p<0.05, **p < 0.01, ***p < 0.001. All data are representative of at least three independent experiments and are presented as the means ± SD.
